# Evaluating ecosystem caps on fishery yield in the context of climate stress and predation

**DOI:** 10.1002/eap.70036

**Published:** 2025-05-12

**Authors:** Alberto Rovellini, André E. Punt, Martin W. Dorn, Isaac C. Kaplan, Meaghan D. Bryan, Grant Adams, Kerim Aydin, Matthew R. Baker, Cheryl L. Barnes, Bridget E. Ferriss, Elizabeth A. Fulton, Melissa A. Haltuch, Albert J. Hermann, Kirstin K. Holsman, Carey R. McGilliard, Elizabeth A. McHuron, Hem Nalini Morzaria‐Luna, Szymon Surma

**Affiliations:** ^1^ School of Aquatic and Fishery Sciences University of Washington Seattle Washington USA; ^2^ Alaska Fisheries Science Center National Oceanic and Atmospheric Administration Seattle Washington USA; ^3^ Northwest Fisheries Science Center National Oceanic and Atmospheric Administration Seattle Washington USA; ^4^ North Pacific Research Board Anchorage Alaska USA; ^5^ Coastal Oregon Marine Experiment Station Oregon State University Newport Oregon USA; ^6^ CSIRO Environment Hobart Tasmania Australia; ^7^ Centre for Marine Socioecology University of Tasmania Hobart Tasmania Australia; ^8^ Cooperative Institute for Climate, Ocean and Ecosystem Studies University of Washington Seattle Washington USA; ^9^ Pacific Marine Environmental Laboratory National Oceanic and Atmospheric Administration Seattle Washington USA; ^10^ Long Live The Kings Seattle Washington USA; ^11^ Visiting Researcher at Northwest Fisheries Science Center National Oceanic and Atmospheric Administration Seattle Washington USA; ^12^ Institute for the Oceans and Fisheries University of British Columbia Vancouver Canada

**Keywords:** Atlantis, climate‐integrated modeling, ecosystem modeling, ecosystem‐based management, optimum yield, predator–prey dynamics

## Abstract

Ecosystem‐based fisheries management strives to account for species interactions and ecosystem processes in natural resource management and conservation. In this context, ecosystem‐wide caps on total fishery catches have been proposed as one tool to manage multispecies fisheries with an ecosystem approach. However, determining effective ecosystem caps is complicated because fish stock production is influenced by environmental conditions, species interactions, and fishing. Consequently, the implementation of ecosystem caps in fisheries management frameworks remains uncommon. We investigated whether ecosystem caps should account for climate variability and for predator–prey dynamics to achieve management objectives in complex marine ecosystems. We considered the example of the Gulf of Alaska (United States), a North Pacific large marine ecosystem where annual groundfish catches are managed using an “optimum yield” ecosystem cap of 800,000 t. We simulated multispecies yield of the 12 most abundant and commercially valuable groundfish stocks under selected climate and fishing scenarios using an end‐to‐end marine ecosystem model (Atlantis), which accounts for predator–prey and ecosystem dynamics. We found that total groundfish yield was never projected to exceed the 800,000 mt optimum yield cap across scenarios and fishing mortalities. Projected climate change led to decreased groundfish yield, and predation from the underexploited groundfish predator arrowtooth flounder ( *Atheresthes stomias* ) led to foregone catches. Groundfish removals had negative indirect effects on groundfish predators, despite total yield never exceeding the optimum yield cap, highlighting that an ineffective cap may not protect non‐target species. These results suggest that the optimum yield cap currently used in the Gulf of Alaska may be too high to constrain groundfish catches under future climate change and low exploitation rates of predators. We propose that ecosystem caps should be reviewed when environmental conditions, stock productivity, or species interactions change.

## INTRODUCTION

Ecosystem‐level caps on total fishery catch (ecosystem caps hereafter) are a limit to total biomass removals from an ecosystem and have been proposed as one tool to manage multispecies fisheries using an ecosystem‐based fisheries management approach (Morrison et al., 2024; Patrick & Link, 2015). Indices based on total catch have been proposed to evaluate the status of marine ecosystems (Link & Watson, 2019), with examples from Africa (Link et al., 2020) and the United States (US; Link, 2021). Similarly, simulated management approaches focusing on total multispecies catch were shown to meet socioeconomic and conservation objectives in Australia (Fulton et al., 2019) and the United States (Gaichas et al., 2017).

Implementation of ecosystem caps in fisheries management is not widespread, although a small number of cases exist. For example, the Northwest Atlantic Fisheries Organization, which manages fishing in the high seas in the northwest Atlantic Ocean, presents estimates of ecosystem‐level yield to managers as ancillary information for decision making (Koen‐Alonso et al., 2019). Thailand's multispecies fishery is managed using the maximum sustainable yield (MSY) of its aggregate marine resources as a limit (Fulton et al., 2022; Kulanujaree et al., 2020). Annual federally managed groundfish catches in North Pacific (US) are limited by a fishery‐wide “optimum yield” (OY) cap. In the United States, OY is defined as the catch that would provide the greatest benefit to the nation while being lower than or equal to MSY (Restrepo et al., 1998). The OY cap was set to 2 million mt in the Bering Sea and 800,000 mt in the Gulf of Alaska (NPFMC, 2019; Witherell, 2000). In the Bering Sea, aggregate catch limits exceeding the OY cap trigger a North Pacific Fishery Management Council (NPFMC) process to reduce single‐species yields. In the Gulf of Alaska, the OY cap was originally defined as the sum of single‐species MSY prior to 1987, reduced by 8% to be more risk averse (Witherell, 2000), but this cap has never constrained groundfish yields in this region (Mueter & Megrey, 2006; NPFMC, 2023).

One difficulty in implementing ecosystem caps is that total fishery yield varies over time depending on primary productivity and energy transfer through the food web (Link & Watson, 2019; Stock et al., 2017). Although trophic interactions influence stock productivity (Gaichas et al., 2012; Reum et al., 2020; Tyrrell et al., 2011), predator–prey dynamics have not typically been considered in tactical management historically (Skern‐Mauritzen et al., 2016). However, predator–prey considerations have increasingly found a role in management frameworks, especially for defining rates of time‐varying natural mortality, considering trophic impacts on single‐species reference points, and providing ecosystem context to fishery managers (Karp et al., 2023). For instance, a few stock assessments account for predation (Howell et al., 2021), for example, by Atlantic cod (*Gadus morhua*, Gadidae) on capelin (*Mallotus villosus*, Osmeridae) in the Barents Sea (Hjermann et al., 2010) and on Norway lobster (*Nephrops norvegicus*, Nephropidae) in the Irish Sea (Bentley et al., 2020). Trophic interactions are considered in an ad hoc manner in the management of fish stocks around Antarctica, for example, by avoiding depletion of Antarctic krill (*Euphausia superba*, Euphausiidae) in areas used by their predators (CCAMLR, 2023). In the Bering Sea, a multispecies model accounting for predation mortality is run alongside the single‐species assessment for walleye pollock (*G. chalcogrammus*) and provides risk‐related information about ecosystem concerns (Holsman et al., 2023); additionally, harvest control rules for some groundfish species are designed to ensure sufficient prey availability for endangered Steller sea lions (*Eumetopias jubatus*, Otariidae; NMFS, 2000). However, existing ecosystem caps have been implemented by summing estimates of single‐species MSY, without accounting for trophic interactions among target species or between these species and their prey and predators (Witherell, 2000). Recent research has highlighted that ecosystem caps would not be expected to be simply the sum of single‐species MSY; instead, they might be ecosystem‐level reference points and be informed by calculations of surplus production, primary production, or by reducing the aggregate single‐species MSY to account for trophic interactions among target species (Morrison et al., 2024).

Climate change further complicates the identification of effective ecosystem caps by affecting ecosystem productivity and species distributions (Gaines et al., 2018; Travers‐Trolet et al., 2020). For example, the 2 million mt OY cap in the Bering Sea is projected to cease constraining groundfish catches around mid‐century, when ocean warming is predicted to lead to declines in groundfish biomass (Holsman et al., 2020).

Aggregate production models have shown that ecosystem‐level MSY is lower than the sum of single‐species MSY and is affected by species interactions and environmental conditions, particularly temperature (Bundy et al., 2012; Fogarty et al., 2012; Mueter & Megrey, 2006). Such production models are tractable and easily interpretable, but more complex models can explicitly include spatiotemporal variability in climate and species interactions. End‐to‐end ecosystem models can provide detailed insight into ecosystem‐level productivity and the indirect effects of multispecies fishing on the prey and predators of target species (Fulton et al., 2022).

**FIGURE 1:**
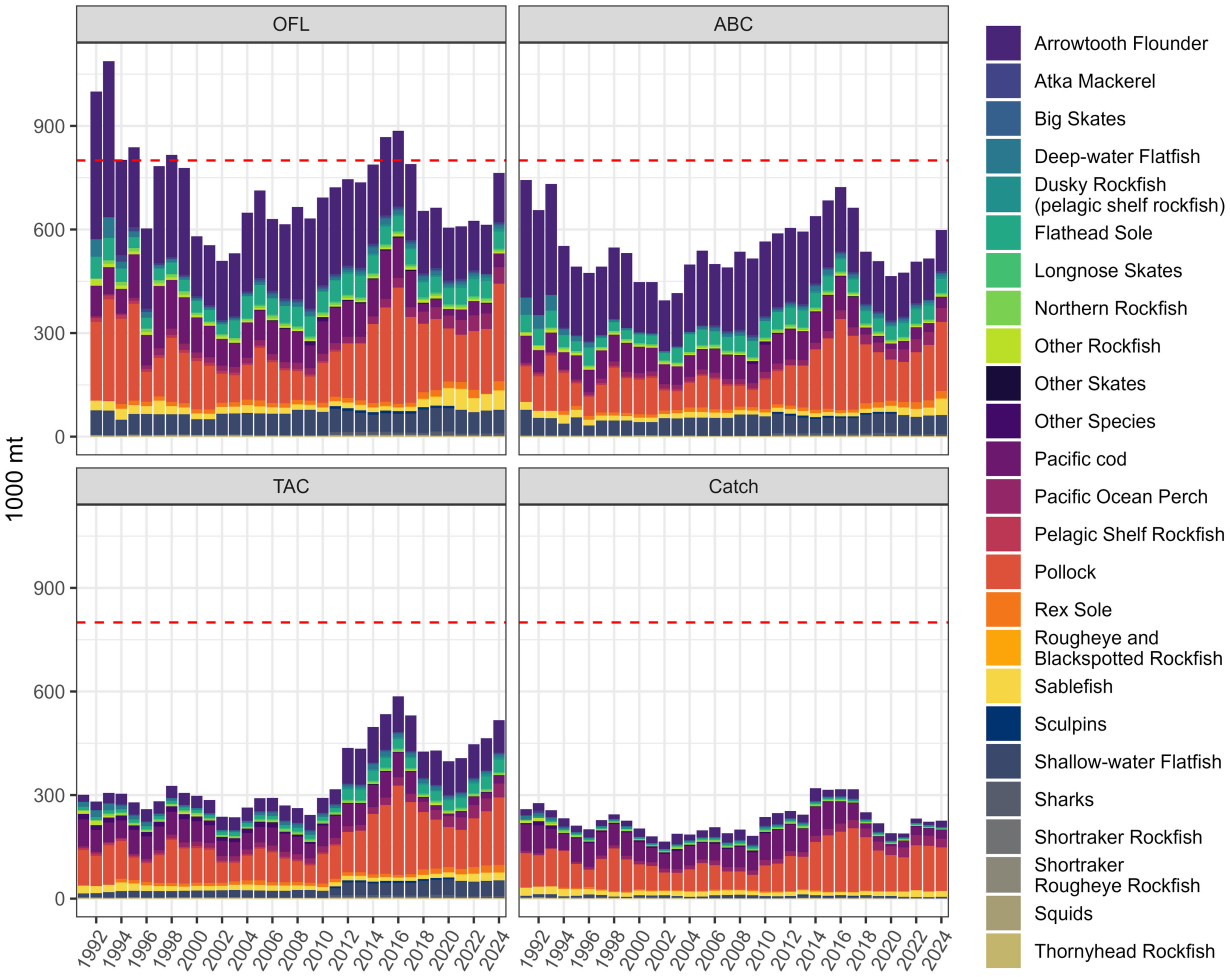
Historical values of the aggregate catch allocation metrics and total catch (sum of landed and discarded) of species included in the Gulf of Alaska (GOA) groundfish Fishery Management Plan. ABC, acceptable biological catch; OFL, overfishing limit; TAC, total allowable catch. OFL is a proxy of maximum sustainable yield (MSY) in Alaska according to current definitions. The horizontal dashed red line is the 800,000 mt optimum yield GOA cap.

In this study, we investigate the effects of climate and predation on total catch in the context of ecosystem caps, with an illustrative example from the Gulf of Alaska (GOA). We apply a climate‐linked end‐to‐end ecosystem model (Atlantis, Fulton et al., 2011) to the GOA, where annually specified aggregate catch limits have never exceeded the 800,000 mt OY cap (Figure 1; Mueter & Megrey, 2006). We explore the ecological tradeoffs that emerge when harvesting trophically linked groundfish stocks under various climate‐fishing scenarios. Under each scenario, we evaluate the effects on the single‐species yield of key stocks, the indirect effects on the biomasses of key groundfish prey and predators as mediated by trophic interactions, and how multispecies groundfish yield relates to the OY cap.

## METHODS

### Case study area: The Gulf of Alaska

The GOA is a productive large marine ecosystem that supports numerous commercially important fisheries, with the gross value of groundfish products totaling USD 248 million in 2021 (Abelman et al., 2023). Past climate events in the GOA have resulted in positive or negative effect on exploited groundfish stocks, for example, a large increase in groundfish recruitment in the 1980s following the 1977 regime shift (Anderson & Piatt, 1999) and increased adult mortality and recruitment failure for Pacific cod (*G. macrocephalus*; Barbeaux et al., 2020) during the 2014–2016 North Pacific marine heatwave (Di Lorenzo & Mantua, 2016).

The GOA features high predator biomass and some degree of top‐down control (Barnes et al., 2020; Gaichas et al., 2015). Arrowtooth flounder (*Atheresthes stomias*, Pleuronectidae; arrowtooth hereafter) is a key predator of commercially important groundfish such as walleye pollock (pollock hereafter) and Pacific cod. Arrowtooth has low market value and has supported limited directed commercial fisheries historically, although recent efforts to derive viable arrowtooth products have motivated some targeted fishing (Shotwell et al., 2023). However, due to its role as a key predator, trends in arrowtooth biomass may affect the biomasses of other stocks and, consequently, total ecosystem yield. The GOA also supports populations of seabirds and marine mammals, including endangered Steller sea lions in the western GOA and Aleutian Islands, which are important predators of groundfish and forage fish.

Annual total groundfish catches in the GOA have always been lower than the aggregate catch allocations for the region due to low attainment for some species (e.g., flatfish stocks) and much lower than the 800,000 mt cap (Figure 1). Since 1992, total GOA groundfish catches have varied between 165,117 mt in 2002 and 321,276 mt in 2014, with a mean of 229,847 mt and a value of 226,001 mt in 2024.

### An ecosystem model to evaluate the GOA OY cap

We apply an Atlantis ecosystem model (“Atlantis GOA”; Rovellini et al., 2024) to evaluate multispecies groundfish yield with respect to the 800,000 mt cap (OY cap hereafter) under various fishing and climate scenarios. Atlantis is a spatially explicit, deterministic simulation model that couples physical, biogeochemical, and socioeconomic sub‐models (Fulton et al., 2011). Atlantis tracks nutrient flows across trophically linked species and functional groups in a 3D model domain using a user‐defined (12‐h for Atlantis GOA) time step and forward difference integration. Physical variables, such as temperature, are dynamically linked to ecological processes, and fishing mortality can be imposed on exploited functional groups (Audzijonyte et al., 2019). The model domain of Atlantis GOA covers the continental shelf down to 1,000 m depth and extends from 170° W along the Aleutian Islands and Alaskan coast to northern British Columbia (Canada) (Appendix S1: Figure S1). The model was initialized to represent the GOA in the early 1990s and forced with the physical conditions and fishing mortality described below. Details of model structure, development, calibration, and skill assessment can be found in Rovellini et al. (2024).

The physical sub‐model captures the key oceanographic features of the GOA and is driven by indices of temperature, salinity, and water transport between spatial cells derived from a coupled Regional Oceanic Modeling System (ROMS) model for the Northeast Pacific at a 10‐km resolution (Coyle et al., 2019). The ROMS model (Haidvogel et al., 2008; Shchepetkin & McWilliams, 2005) utilizes a curvilinear horizontal grid, with terrain‐following vertical coordinates and 42 vertical levels. The hindcast run, which spans 1990–2020, utilized atmospheric forcing and oceanic boundary conditions from the NCEP Climate Forecast System, as described in Coyle et al. (2019). Here, we forced Atlantis using ROMS‐derived indices for 1999. We chose this year for model calibration because it was the first year of available ROMS output that was not characterized by El Niño conditions in the GOA (Stabeno et al., 2004), representing conditions prior to the onset of the 2014–2016 marine heatwave. Heatwave years were denoted by anomalous biophysical conditions in the GOA that we did not simulate in this study, but model responses to 2014 temperature and food availability have been explored by Rovellini et al. (2024). We repeated the 1999 physical forcing in a loop to eliminate interannual variability while maintaining seasonal variation in the physical forcings (Rovellini et al., 2024).

The GOA food web is represented as 78 functional groups (see file Atlantis_GOA_functional_groups.xlsx in Rovellini, 2025), with vertebrates modeled as age‐structured populations and invertebrates as biomass pools. Ontogenetic, spatially explicit trophic interactions were parameterized using multiple data sources (Rovellini et al., 2024), with groundfish diets informed by stomach content data (Livingston et al., 2017). For example, Appendix S1: Figure S2 shows age‐specific diet compositions for arrowtooth in the calibrated Atlantis GOA model. Benthic invertebrates, pelagic zooplankton, and Pacific capelin (*M. catervarius*, capelin hereafter) are important for younger age classes, whereas pollock, Pacific cod, and other groundfishes (including younger arrowtooth) are important for older age classes. Appendix S1: Figure S2 agrees with observations summarized in Doyle et al. (2018).

Temperature is linked to ecological processes in Atlantis GOA in three main ways (Rovellini et al., 2024). First, thermal tolerance niches restrict species spatial distributions; second, spawning occurs within species‐specific temperature ranges to capture stenothermic reproduction of some GOA species (e.g., Pacific cod, Laurel & Rogers, 2020); and third, ectotherm consumption is scaled following a unimodal bioenergetic response (sensu Kitchell et al., 1977). In addition, indirect responses to temperature can be captured in Atlantis by simulating, for example, decreased plankton productivity and food availability, which can impair growth (sensu Rovellini et al., 2024).

Fishing in Atlantis GOA is modeled using species‐specific, time‐invariant fishing mortality *F* (Audzijonyte et al., 2019). For model calibration, stocks were fished at ¼ *F*_OFL_, where the OFL is the overfishing limit, defined as the catch above which overfishing occurs (NPFMC, 2019). *F*_OFL_ is estimated in single‐species stock assessments and defined as the fishing mortality level that, if exceeded, would correspond to the stock being overfished (NPFMC, 2019). *F*_OFL_ is a proxy for *F*_MSY_ (i.e., the *F* that would achieve MSY) in the North Pacific management system (NPFMC, 2019). A value of ¼ *F*_OFL_ was chosen to represent a moderate fishing mortality because several GOA stocks, including all federally managed flatfishes, are exploited below *F*_OFL_ (NPFMC, 2019). In the GOA, estimates of *F*_OFL_ are only available for stocks that have reliable point estimates of biomass and *F*_MSY_ proxies (NPFMC, 2019). For functional groups where *F*_OFL_ was not available, *F* was set to ¼ natural mortality (*M*). *M* values were taken from single‐species stock assessments where available; otherwise, they were computed based on the species' age structure in Atlantis. *F*_OFL_ for the stocks within multispecies functional groups was averaged.

The implementation of Atlantis applied here differs from that in Rovellini et al. (2024) in two main ways. First, we included unimodal bioenergetic responses of consumption to temperature for all fish functional groups (Appendix S1: Table S1), using the formulation from Holsman and Aydin (2015), parameterized by conducting a literature review as detailed in Rovellini et al. (2024). Second, we set the age at 50% fishery selectivity to align better with the age at first maturity, to avoid excessive stock productivity that arises when the youngest spawning age classes are below the age at selectivity. This was necessary for some of the exploited functional groups (particularly the longer‐lived species represented using multi‐year age classes) because fishery selectivity in Atlantis was assumed to be a knife‐edged function based on the first age class fished, whereas maturity is represented with an ogive (Audzijonyte et al., 2019).

### Fishing simulation experiment

We designed a fishing simulation experiment that involved 12 groundfish functional groups (“focal groups” hereafter), chosen among the 78 functional groups in the model because of their ecological and socioeconomic importance (Table 1). These included 11 stocks that, together, typically constitute >90% of the total federally managed groundfish catch in the GOA (NPFMC, 2023). In addition, we included Pacific halibut (*Hippoglossus stenolepis*, Pleuronectidae; halibut hereafter) as the 12th focal group because of its commercial importance and ecological role as a predator of groundfish in the GOA (Barnes et al., 2020), as shown also by model output diet composition for this species (Appendix S1: Figure S3). A directed longline fishery for halibut in the northeast Pacific is managed by the International Pacific Halibut Commission, whereas halibut bycatch in the GOA is managed by the NPFMC; as such, halibut catches do not count toward the OY cap.

**TABLE 1:** Groundfish functional groups in Atlantis Gulf of Alaska (GOA), with corresponding species, summary of their mean annual catch by decade, and values of fishing mortality at the overfishing limit (*F*_OFL_) from single‐species stock assessments when available.

Atlantis group	AKFIN species/complex	Species name	1990–1999	2000–2009	2010–2019	2020–2024	*F*_OFL_
Focal species							
Arrowtooth flounder	Arrowtooth flounder	*Atheresthes stomias*	18,614 ± 3343	23,878 ± 4622	24,545 ± 5722	14,046 ± 5355	0.23
Deep‐water flatfish	Deep‐water flatfish	*Microstomus pacificus*	4581 ± 3110	614 ± 237	293 ± 131	106 ± 15	0.11
Flathead sole	Flathead sole	*Hippoglossoides elassodon*	2136 ± 719	2640 ± 688	2552 ± 556	938 ± 585	0.25
Pacific Ocean Perch	Pacific Ocean Perch	*Sebastes alutus*	6667 ± 3082	11,843 ± 1122	19,148 ± 4726	27,748 ± 2260	0.12
Pacific cod	Pacific cod	*Gadus macrocephalus*	65,800 ± 10,127	50,569 ± 5691	60,990 ± 26,502	19,600 ± 8000	0.51
Rex sole	Rex sole	*Glyptocephalus zachirus*	3773 ± 1162	3030 ± 875	2482 ± 904	642 ± 365	0.3
Rockfish—pelagic shelf assemblage	GOA dusky rockfish	*S. variabilis*			3044 ± 478	2681 ± 544	0.11
	Black/blue rockfish	*S. melanops, Sebastes mystinus*	230 ± 72	228 ± 34			
	Pelagic shelf rockfish		3032 ± 753	3059 ± 489	2841 ± 425		
Rockfish—slope assemblage	Northern rockfish	*Sebastes polyspinis*	3896 ± 1985	4154 ± 735	3590 ± 1050	1823 ± 581	0.06
	Shortraker/rougheye rockfish	*S. borealis, Sebastes aleutianus*	1755 ± 374	1188 ± 309	1351 ± 232	876 ± 126	
	Other slope rockfish		3090 ± 3372	450 ± 147			
Sablefish	Sablefish	*Anoplopoma fimbria*	17,946 ± 4453	13,272 ± 1270	11,058 ± 1068	16,102 ± 2434	0.09
Shallow‐water flatfish	Shallow‐water flatfish	*Lepidopsetta polyxystra*, *L. bilineata*	6210 ± 2666	6736 ± 2069	3947 ± 1129	2287 ± 1364	0.25
Walleye pollock	Pollock	*Gadus chalcogrammus*	92,596 ± 21,895	60,239 ± 12,484	128,311 ± 40,031	118,270 ± 15,117	0.31
Other FMP groundfish							
Big skate	GOA skate, big	*Beringraja binoculata*		1540 ± 385	1900 ± 460	1049 ± 198	
Longnose skate	GOA skate, Longnose	*Raja rhina*		1215 ± 195	1265 ± 322	929 ± 210	
Other skate	GOA skate, other	*Bathyraja aleutica, B. interrupta, Bathyraja parmifera*		1288 ± 298	1461 ± 402	685 ± 186	
	Shark				1719 ± 849	1797 ± 368	
Large sculpins	Sculpin	*Hemilepidotus jordani, Hemitripterus bolini, Myoxocephalus polyacanthocephalus, Myoxocephalus jaok*			1085 ± 434		
Rockfish—demersal shelf	Demersal shelf rockfish	*Sebastes ruberrimus, Sebastes babcocki*	638 ± 181	901 ± 377	1179 ± 123	1149 ± 297	
Shallow demersal fish	Atka mackerel	*Pleurogrammus monopterygius*	1833 ± 2,013	921 ± 784	1362 ± 410	654 ± 248	
Thornyhead	Thornyhead rockfish	*Sebastolobus* spp.	1283 ± 323	883 ± 177	923 ± 226	292 ± 116	
	Other species		5630 ± 3203	4133 ± 1931			
Total							
Total focal species			228,482 ± 20,682	181,425 ± 11,823	261,271 ± 39,878	204,119 ± 20,841	
Total FMP groundfish			237,866 ± 24,122	190,569 ± 12,451	272,076 ± 40,264	211,790 ± 21,007	

*Note*: Pacific halibut is not included in this table because it is not included in the GOA groundfish Fishery Management Plan (FMP). Halibut *F*_OFL_ was assumed to correspond to natural mortality *M* = 0.2 year^−1^ (Stewart & Hicks, 2022). Values of annual catch by decade are mean ± standard deviation. Data used to create this table was obtained from the Alaska Fisheries Information Network (AKFIN).

The fishing experiment proceeded in two steps. In Step 1, the 12 focal groups were exposed to varying fishing mortality one focal group at a time, with the purpose of determining *F*_MSY_ for each focal group (sensu Walters et al., 2005). In Step 2, we manipulated fishing mortality on all focal groups simultaneously by applying multipliers to the vector of 12 *F*_MSY_ values obtained in Step 1.

### Step 1: Manipulating fishing mortality one focal group at a time

**FIGURE 2:**
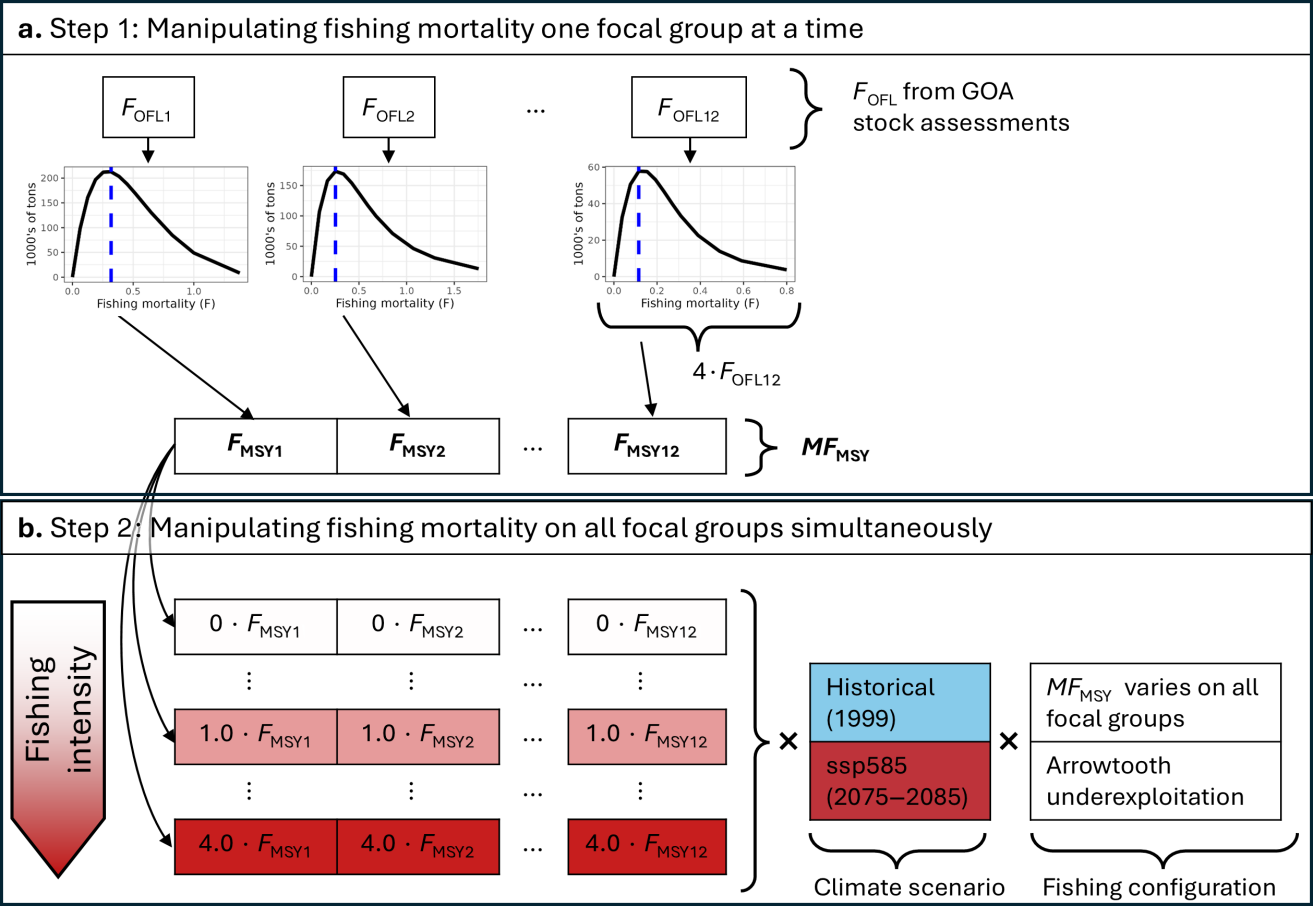
Schematic representation of the fishing experiments performed on the 12 focal groups. Steps 1 and 2 are represented in panels (a) and (b), respectively. *F*_OFL_ is the fishing mortality at the overfishing limit from single‐species stock assessments for the GOA, and a value of 4 *F*_OFL_ was used as the maximum fishing mortality (*F*) explored in Step 1 for each focal group. *F*_MSY_ is the fishing mortality at maximum sustainable yield obtained in Atlantis from profiling *F*, one focal group at a time. *MF*_MSY_ is the vector of the 12 *F*_MSY_ values, one per focal group.

We conducted a series of simulations to determine *F*_MSY_, that is, the *F* that led to the highest equilibrium catch, for each focal group (Figure 2a). To do so, we profiled *F* for one focal group at a time over 13 values between 0 and 4 times the *F*_OFL_ from single‐species assessments (NPFMC, 2019). This was a compromise that allowed us to explore a broad range of fishing intensities despite the computing costs of running long Atlantis simulations. We fixed *F* at ¼ *F*_OFL_ for all other groups (sensu Rovellini et al., 2024). This stock‐specific catch at *F*_MSY_ is not expected to equate to MSY estimates from single‐species assessments because species interactions are present in Atlantis. We chose 4 *F*_OFL_ as the maximum *F* to evaluate productivity under high fishing pressure, a level of fishing substantially larger than the limits that could legally be imposed by management as well as the historical values of *F* for these stocks. This approach allowed us to explore *F* proportionately to stock productivity. This design resulted in 156 model runs (13 *F* values for 12 focal groups).

The purpose of this step was to characterize productivity and determine *F*_MSY_ for these stocks under historical biophysical conditions. Forcing the model with different climate scenarios in this simulation step would likely have led to different estimates of *F*_MSY_ for these stocks (Travers‐Trolet et al., 2020). However, we elected to focus on the effects of climate on catch and stock productivity in Step 2 (presented below), which better captures multispecies fishing.

We present all results as mean values at equilibrium over the last five years of each simulation run. We ran all simulations for 80 years, inclusive of a 30‐year burn‐in period with fishing and physical forcings set to the calibration values (i.e., ¼ *F*_OFL_ and 1999 physics, respectively). The purpose of the burn‐in period is to allow the model to cycle through initial instabilities (Pethybridge et al., 2019). The total duration of 80 years has been observed to be sufficiently long for the base model to approach equilibrium under calibration conditions (Rovellini et al., 2024), although forcing the model with climate or fishing scenarios other than the calibration ones may lead to different end‐of‐run equilibrium conditions.

In this step, we extracted terminal annual catch at equilibrium as an average of the last five years from each run and the corresponding CVs. We then selected the *F* that returned the highest catch as the *F*_MSY_ for that focal group and combined the *F*_MSY_ for each of the 12 focal groups into a vector, denoted *MF*_MSY_.

### Step 2: Manipulating fishing mortality on all focal groups simultaneously

We developed four scenarios that combined climate regime and arrowtooth exploitation rates (Figure 2b). Simulation duration and burn‐in period were identical to Step 1. The four scenarios, each applying 13 levels of fishing mortality, were as follows:

1. *MF*_MSY_ varies for all focal groups, historical climate conditions. We multiplied the vector of *MF*_MSY_ from Step 1 by 13 scalars ranging from 0 to 4. We chose 4 as the maximum value to reach a fishing pressure comparable to the values used for Step 1, but for all 12 focal groups at once. Fishing mortality on all other functional groups remained at calibration levels of ¼ M (Rovellini et al., 2024), to better identify the effect of varying fishing intensity on the 12 focal groups. As in Step 1, we forced this scenario with physics for the year 1999.
2. Arrowtooth underexploitation, *MF*_MSY_ varies for all other focal groups, historical climate conditions. This scenario was identical to the first, except that *F* on arrowtooth remained at the calibration level of ¼ *F*_OFL_. This allowed for the exploration of cases where all GOA groundfishes are heavily exploited (i.e., for the larger multipliers of *MF*_MSY_) and arrowtooth is fished at a lower intensity. This scenario aimed to explore the effects on yield when a predator that has limited commercial value is lightly exploited (Doyle et al., 2018) but is also expected to exert top‐down control on other stocks (Adams et al., 2022; Barnes et al., 2020), as is currently the case for arrowtooth in the GOA.
3. *MF*_MSY_ varies for all focal groups, projected climate change. This scenario was identical to the first, but with physical forcings representing future climate change conditions (Hicke et al., 2022). Alaska groundfishes respond to climate drivers (e.g., increased temperature and decreased plankton productivity) in complex ways, including altered weight‐at‐age and impaired recruitment (Barbeaux et al., 2020; Laurel et al., 2016; Oke et al., 2022). Atlantis GOA captures these sensitivities and can therefore be used to evaluate ecosystem‐level responses to changes in temperatures. We forced temperature, salinity, and water transport with ROMS‐derived indices for 2075–2085 from a high carbon emission scenario (ssp585 from the GFDL‐ESM4 projection, developed as part of the Coupled Model Intercomparison Project Phase 6; Dunne et al., 2020). The ROMS configuration used for scenario downscaling is identical to that used for hindcasting but is instead driven with atmospheric forcing, oceanic boundary conditions, and coastal runoff derived from the GFDL “historical” and ssp585 output, which span 1980–2014 and 2015–2100, respectively. For use in Atlantis, we corrected projected temperature and salinity indices $x_{c,t}^{\text{proj}}$ for each model cell *c* and at daily time steps *t* with a delta method, to account for bias in the GFDL model, with the formula:
(1) $$x_{c,t}^{\text{proj}\prime }={\bar{x}}_{c,T}^{\text{hind}}+\left(x_{c,t}^{\text{proj}}-{\bar{x}}_{c,T}^{\text{hist}}\right),$$
  where $x_{c,t}^{\text{proj}\prime }$ is the delta‐corrected value, ${\bar{x}}_{c,T}^{\text{hind}}$ is the average index value from the ROMS hindcast driven by atmospheric and oceanic reanalysis products for the reference period *T* (1991–2014), and ${\overset{¯}{x}}_{c,T}^{\text{hist}}$ is the average index value from the free‐running ROMS historical run over *T*. Then, for each spatial cell, we calculated daily climatologies of temperature and salinity for 2075–2085, which yielded one year's worth of physical forcings. This single year was used in a loop to force the model for 50 years (after 30 years' burn‐in under 1999 conditions). The purpose of using one year of daily climatologies, instead of the original sequence of ROMS outputs for 2075–2085, was to minimize interannual variability in the physical forcings, thereby reflecting equilibrium conditions while capturing seasonality and spatial patterns. Increased temperature in the GOA is associated with decreased low‐trophic level productivity (Batten et al., 2018; Piatt et al., 2020; von Biela et al., 2019); so, we also halved the growth rates and productivity of diatoms, copepods, and euphausiids (sensu Rovellini et al., 2024). Sensitivity of the model to this assumption was evaluated in Rovellini et al. (2024), who found that forcing decreased plankton productivity was necessary to capture the bottom‐up effects of food limitation on forage fish observed in the GOA under warm conditions (Arimitsu et al., 2021).
4. Arrowtooth underexploitation, *MF*_MSY_, varies for all other focal groups, projected climate change. This scenario combined arrowtooth fishing from the second scenario with climate conditions from the third scenario. The purpose of this scenario was to explore the combined effects of arrowtooth predation and warming on the GOA ecosystem and fisheries.

We evaluated each simulation in terms of annual catch, spawning stock biomass, and numbers‐at‐age for the focal groups, averaged over the final five years of the projection period. We also examined biomasses of key forage fish species and selected piscivorous predators relative to changes in the total biomass of their predator and prey species, respectively, to evaluate the indirect effects of groundfish removals on the food web. The forage fish functional groups we considered were capelin, Pacific sand lance (*Ammodytes personatus*, Ammodytidae), Pacific herring (*Clupea pallasii*, Clupeidae), eulachon (*Thaleichthys pacificus*, Osmeridae), and a multispecies group for slope forage fish (Myctophidae and Bathylagidae). The piscivorous predator functional groups we considered were Steller sea lions, other pinnipeds (comprising mostly harbor seals *Phoca vitulina*, Phocidae), dolphins and porpoises (Pacific white‐sided dolphins, *Lagenorhynchus obliquidens*, Delphinidae, and harbor porpoises, *Phocoena phocoena* and Dall's porpoises *Phocoenoides dalli*, Phocoenidae), and two multispecies functional groups of piscivorous seabirds (surface‐feeding and diving). All analyses were performed in R 4.4.0.

## RESULTS

### Step 1: Manipulating fishing mortality one focal group at a time

**FIGURE 3:**
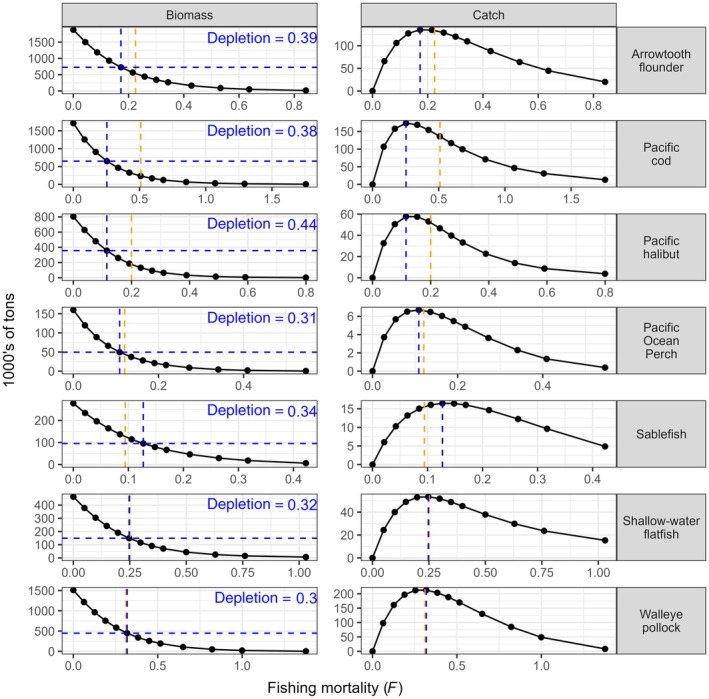
Equilibrium spawning biomass (left) and catch (right) in 1000s of tons for selected focal groups as a function of fishing mortality *F*, as determined in Step 1 of the Atlantis fishing experiment (*F* manipulated on one focal group at a time). Vertical dashed lines: in orange, *F* at the overfishing limit (*F*_OFL_) from single‐species assessments (natural mortality M for Pacific halibut), and in blue, *F* at maximum sustainable yield (*F*_MSY_) as determined by Atlantis. Horizontal dashed lines indicate stock biomass at *F*_MSY_, with the corresponding depletion (i.e., the fraction of unfished biomass).

Varying *F* for one focal group at a time while holding *F* at ¼ *F*_OFL_ for the other focal groups resulted in Atlantis *F*_MSY_ estimates lower than the single‐species stock assessment values of *F*_OFL_ (which are proxies for *F*_MSY_ in the Alaska management system), except for sablefish (*Anoplopoma fimbria*, Anoplopomatidae) and the slope rockfish assemblage. Results for selected groups of high abundance and commercial relevance are shown in Figure 3; all focal groups appear in Appendix S1: Figure S4. The yields of some focal groups (e.g., sablefish) across the explored range of *F* presented relatively broad peaks, with similar yields achieved for a wide range of fishing mortalities. Spawning stock biomass at *F*_MSY_ in Atlantis was between 27% (rex sole *Glyptocephalus zachirus*, Pleuronectidae) and 44% (halibut) of unfished spawning biomass (mean 33%, median 31%). For comparison, the fraction of unfished biomass corresponding to *F*_OFL_ in the single‐species GOA stock assessments is assumed to be 35% (NPFMC, 2019). Production curves showed that most of these stocks exhibited high enough productivity to support some yield even at very low stock status under the highest fishing pressure (4 *F*_OFL_, Appendix S1: Figure S5).

There was general agreement between *F*_MSY_ values obtained here and *F*_OFL_ estimates calculated in the assessments, with the largest discrepancies for Pacific cod, halibut, and flathead sole (*Hippoglossoides elassodon*, Pleuronectidae). End‐of‐run variability of catch and biomass was small for all 12 focal groups but increased with *F*, highlighting that high fishing intensity led to model instability (Appendix S1: Figure S6).

### Step 2: Manipulating fishing mortality on all focal groups simultaneously

**FIGURE 4:**
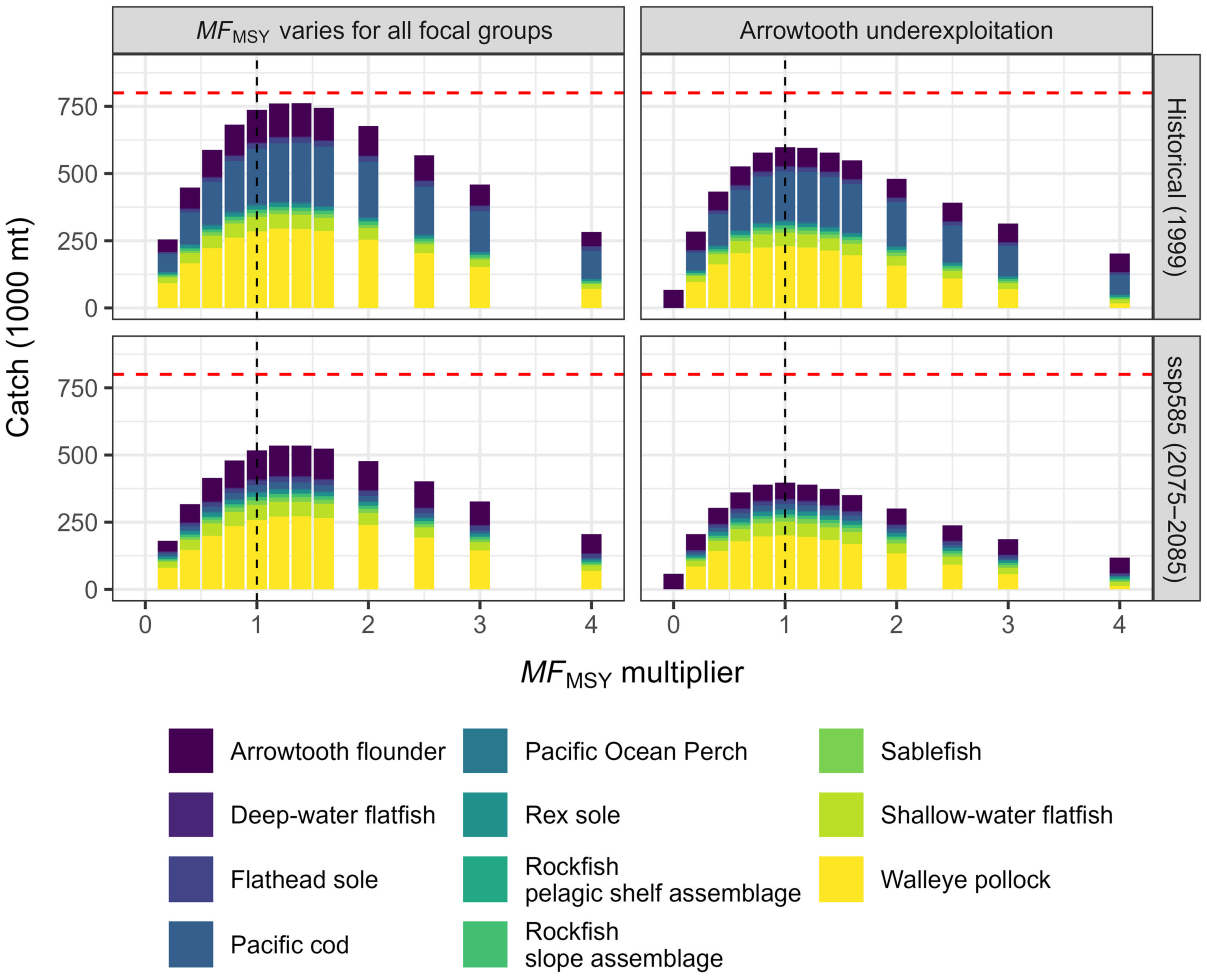
Equilibrium multispecies yield (1000s of tons) of groundfish focal groups for increasing multipliers of the multispecies fishing mortality at maximum sustainable yield (*MF*_MSY_). Panels indicate the four scenarios combining climate regime (rows) and arrowtooth flounder exploitation level (columns). Only catches from the Alaska portion of the model domain are shown, and Pacific halibut catches are not plotted, to allow comparison with the Gulf of Alaska optimum yield cap (horizontal red dashed line = 800,000 t). The vertical dashed line indicates *MF*_MSY_.

**FIGURE 5:**
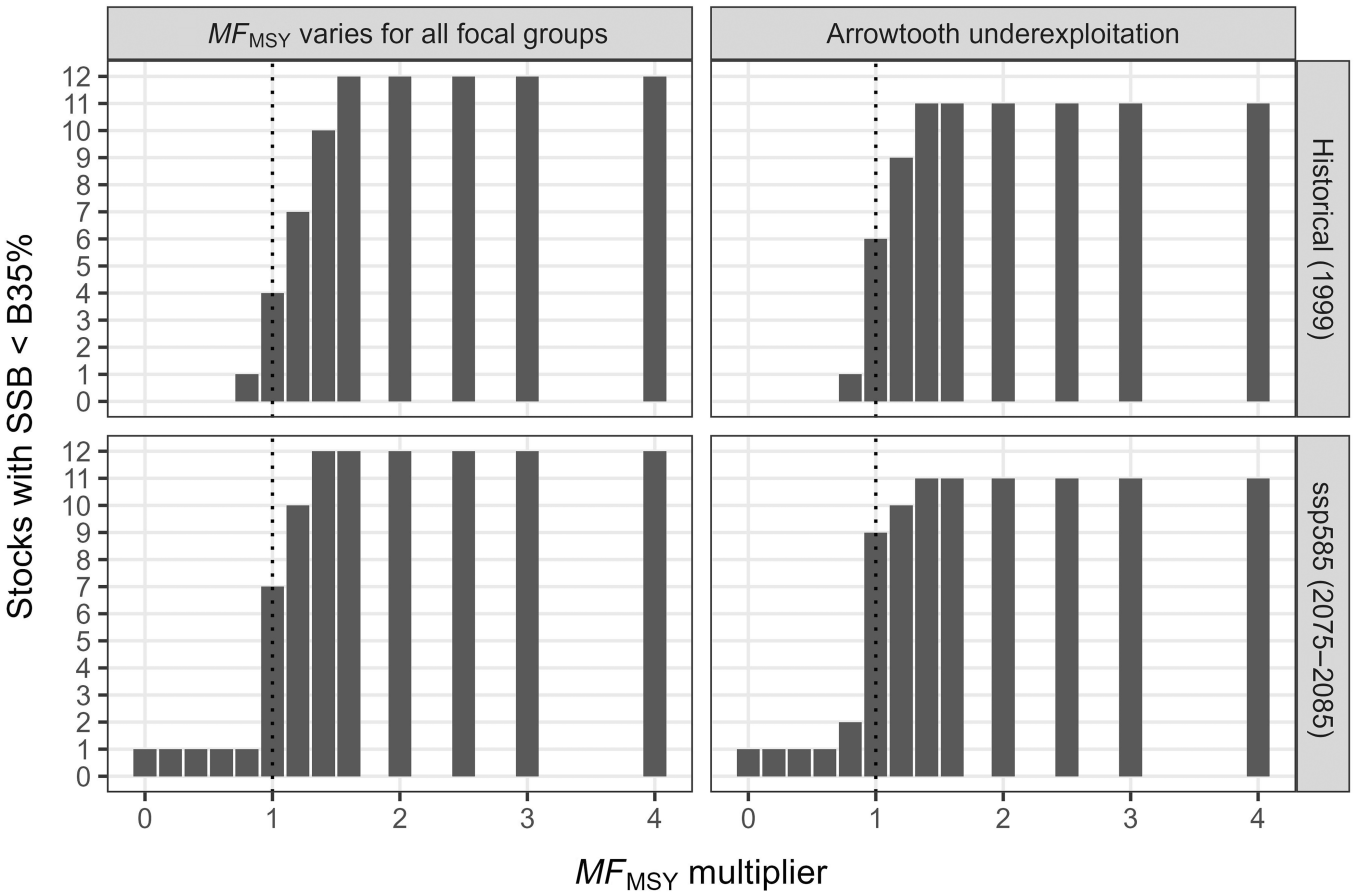
Number of groundfish focal groups (out of 12) whose spawning stock biomass (SSB) fell below B_35%_ (the reference point for overfishing in the North Pacific) at equilibrium for increasing multipliers of the multispecies fishing mortality at maximum sustainable yield (*MF*_MSY_). Panels indicate the four scenarios related to climate regime (rows) and arrowtooth flounder fishing strategy. The vertical dotted line indicates *MF*_MSY_. Note that Pacific cod is below B_35%_ regardless of fishing mortality and arrowtooth fishing strategy under warm climate conditions.

The aggregate yield of the focal groups (less that for halibut catch because this species does not count toward the OY cap under current regulations) never reached the OY cap of 800,000 mt regardless of fishing intensity, climate regime, and arrowtooth exploitation level (Figure 4). However, total catch at *MF*_MSY_ under historical climate and full arrowtooth exploitation was comparable to historical estimates of aggregate MSY (Figure 1). Both projected climate change and arrowtooth underexploitation led to consistently lower aggregate yield of the focal groups, with the scenario combining these two factors returning the lowest yield. The OY cap was reached only under historical climate and full arrowtooth exploitation at or just above *MF*_MSY_ when all groundfish groups were used to compute aggregate yield (Appendix S1: Figure S7). *MF*_MSY_ multipliers ≥1 (i.e., *F* greater than or equal to the *MF*_MSY_ vector obtained from Step 1) caused 9 out of 11 focal groups (i.e., not including arrowtooth) to fall below B_35%_ (the NPFMC reference point for overfishing) at *MF*_MSY_ for the scenario combining projected climate change and underexploited arrowtooth (Figure 5).

The maximum catch at equilibrium of the groundfish focal groups was higher in simulations where *F* was manipulated on all focal groups simultaneously (Step 2) than in those where *F* was profiled one group at a time (Step 1) (Appendix S1: Figure S8). This effect varied among species, with pollock, flathead sole, and Pacific cod having the largest increase in yield when arrowtooth was fished at the same intensity as all other focal groups (34%, 28%, and 24%, respectively). Changes in equilibrium yield were smaller for higher predators (e.g., halibut, sablefish, and arrowtooth) and pelagic species (e.g., Pacific ocean perch *Sebastes alutus*, Sebastidae).

**FIGURE 6:**
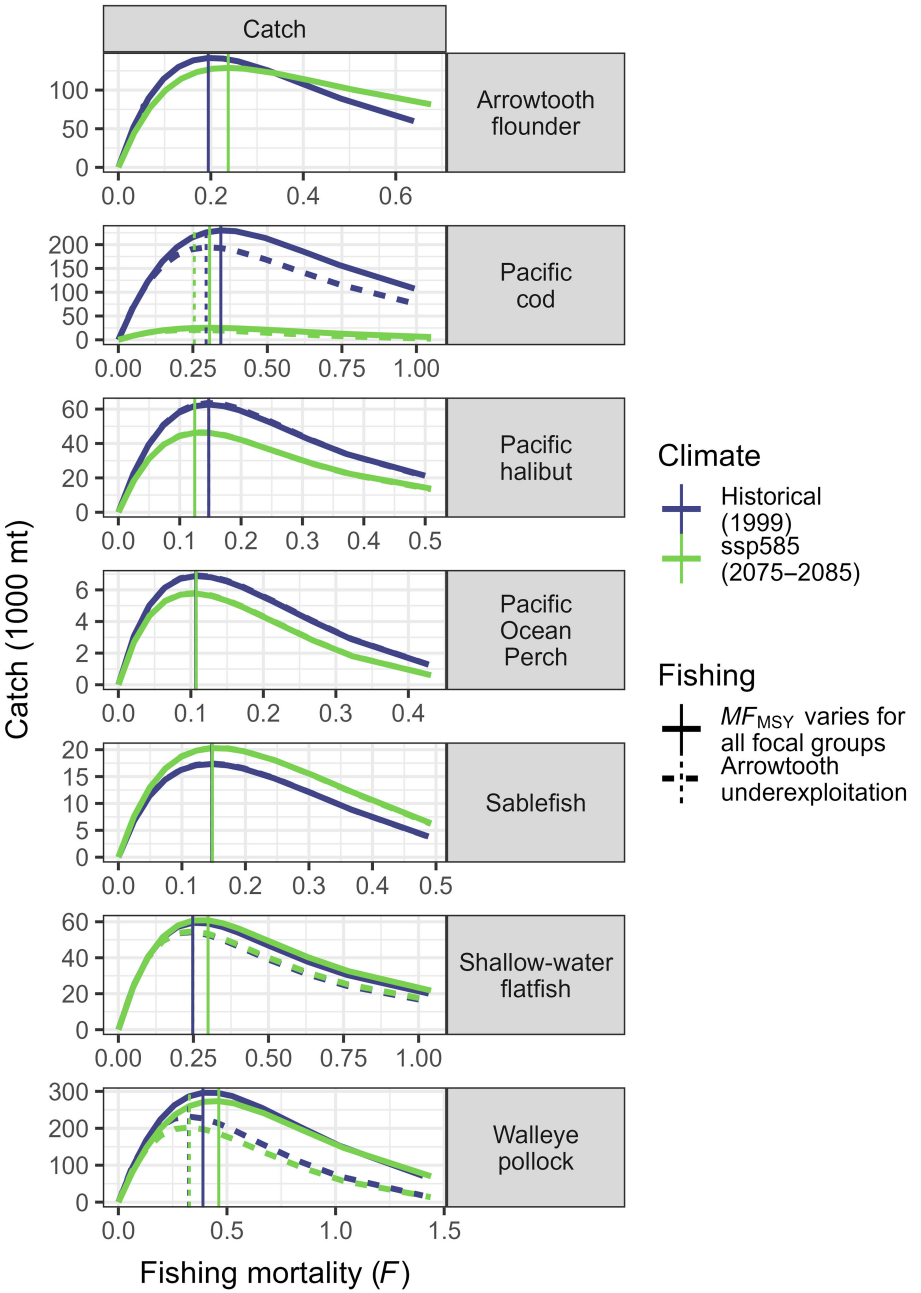
Equilibrium catch (1000s of tons) of selected focal groups for increasing fishing mortality *F* under different combinations of climate regimes and arrowtooth exploitation level. Blue lines: historical conditions (1999); green lines: warm conditions (2075–2085 climatology, ssp585). Solid lines: *F* on all stocks varies with the multipliers of the multispecies fishing mortality at maximum sustainable yield (*MF*_MSY_); dashed lines; *F* on arrowtooth fixed at ¼ *F*_OFL_, a proxy for *F*_MSY_ in Alaska. Vertical lines indicate the *F* corresponding to the highest yield in each scenario.

Focal groups showed variable responses to different combinations of climate regime and arrowtooth exploitation level (Figure 6; Appendix S1: Figure S9). Projected climate change led to generally small effects on catch, irrespective of fishing mortality and arrowtooth exploitation level. The one exception was Pacific cod, which exhibited near complete collapse caused by poor recruitment during warm conditions. Responses of equilibrium catch to arrowtooth exploitation level were stronger for focal groups that are predated on by arrowtooth in Atlantis GOA, including pollock, Pacific cod, flathead sole, shallow‐water flatfish, rex sole, and slope assemblage rockfish.

Biomass and catch CVs over the last five years increased with fishing mortality for most species, with pollock increasing the most (Appendix S1: Figure S10A). Runs simulating projected climate change and runs simulating arrowtooth underexploitation led to higher end‐of‐run variability, with the combination of these two factors resulting in the largest CVs (up to 0.08; Appendix S1: Figure S10B).

Numbers‐at‐age of GOA groundfish decreased with increasing *MF*_MSY_ multiplier (Appendix S1: Figure S11). Responses varied among scenarios, but generally, arrowtooth exploitation level had larger effects on numbers‐at‐age than climate regime, except for Pacific cod and halibut, where numbers‐at‐age were lower under projected climate change. Numbers‐at‐age for some stocks were unaffected by arrowtooth *F*, including halibut, sablefish, deep‐water flatfish, and Pacific ocean perch. Arrowtooth diet compositions at the highest *MF*_MSY_ multiplier were similar across scenarios (Appendix S1: Figure S12) and always contained less groundfish (especially pollock) than arrowtooth diets in the base model exposed to calibration fishing.

### Effects of fishing on key groundfish prey and predators

**FIGURE 7:**
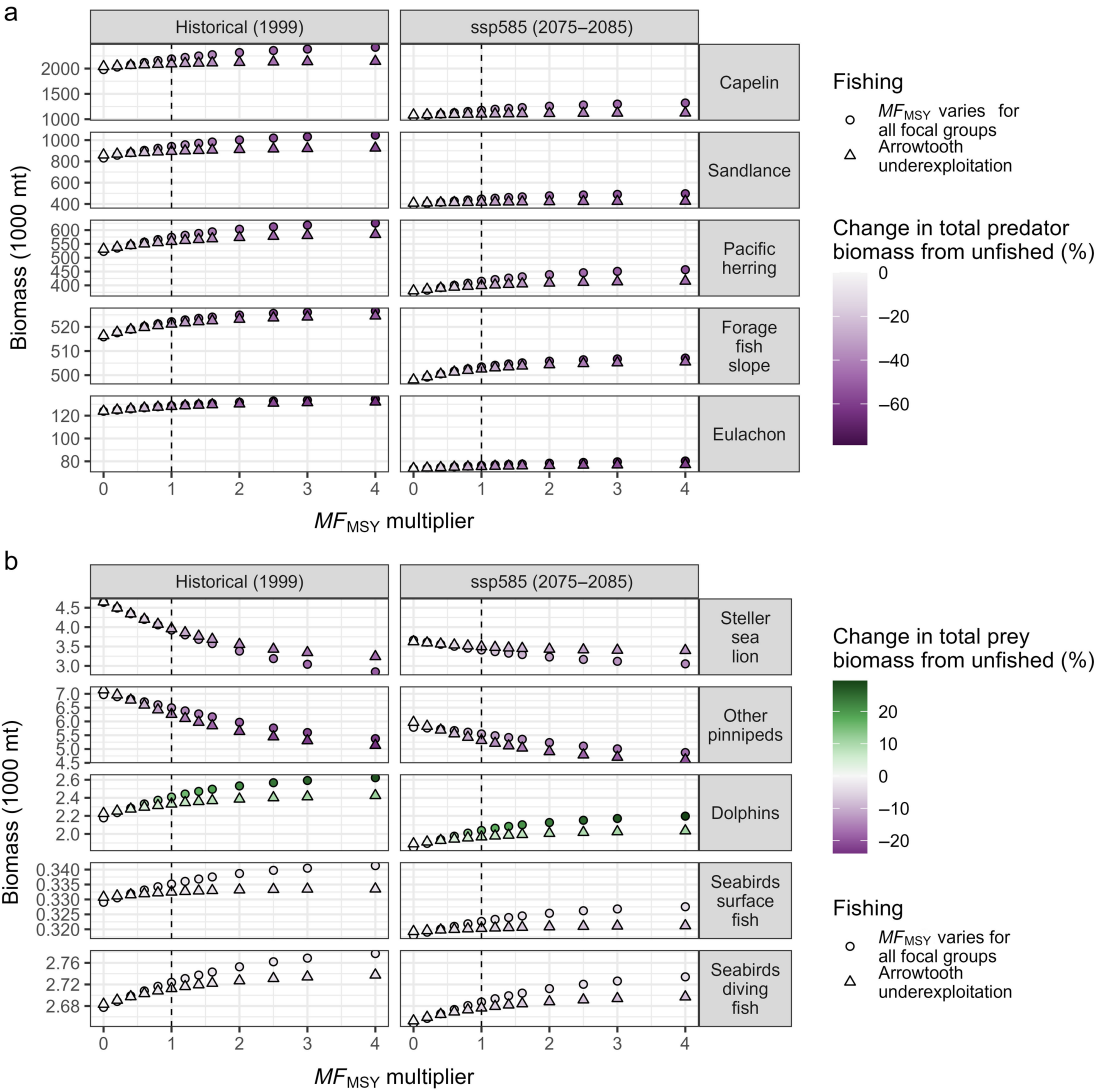
(a) Forage fish biomass (1000s of tons) for increasing multipliers of the multispecies fishing mortality at maximum sustainable yield (*MF*_MSY_) for groundfish. Note the different scales on the *y*‐axis and that the y‐axis does not extend to 0. Colors indicate the change (in percentage) in total biomass of the predators of each forage fish species from the unfished scenario. Shapes indicate the arrowtooth exploitation level, and columns indicate the climate scenario. The vertical dashed line indicates *MF*_MSY_. (b) Same as (a) but for predator biomass (1000s of tons). Colors indicate the change (in percentage) in total biomass of the prey of each piscivorous predator functional group from the unfished scenario.

Removals of groundfish from the system (i.e., increasing *MF*_MSY_ multipliers) led to higher forage fish biomass, although this was lower under projected climate change (Figure 7a). Arrowtooth underexploitation and the consequently higher arrowtooth biomass reduced the benefits to forage fish of exploiting other groundfishes. Responses of groundfish predators, such as pinnipeds, birds, and dolphins and porpoises, to increasing *MF*_MSY_ multipliers (and the resulting depleted groundfish stocks) varied and appeared to be driven by prey dynamics (Figure 7b; Appendix S1: Figure S13). The biomasses of all predator functional groups declined under projected climate change. Pinniped biomass declined with growing groundfish fishing intensity and the resulting decline in total prey biomass. For Steller sea lions, the negative effects of groundfish removals were partially offset by high arrowtooth biomass (which they also eat) under arrowtooth underexploitation. Conversely, dolphin and porpoise biomass increased with higher *MF*_MSY_ multipliers in response to increased availability of capelin and small demersal fishes, which are important prey for this group. Piscivorous seabird biomass varied little among scenarios. Changes in top predator biomass under increased groundfish exploitation were driven largely by changes in the weight‐at‐age of these predators, which highlights that such biomass changes were mediated by trophic interactions.

Biomass CVs over the last five years for forage fish remained constant for increasing values of groundfish *F*, but they increased under warm conditions. CVs of piscivorous predator biomass became more variable for increasing groundfish fishing mortality, suggesting that the end‐of‐run instability of some of the groundfish biomass under high *F* propagates to their predators (Appendix S1: Figure S14).

## DISCUSSION

We show that ecosystem caps can be misspecified if climate variability and species interactions are not considered. We found that the ecosystem cap currently used for the GOA (800,000 mt) is unlikely to constrain groundfish catches under projected climate change and underexploitation of an important predator (arrowtooth). For the GOA, we show that projected climate change leads to decreased groundfish yield and predation from arrowtooth results in foregone groundfish catches, irrespective of fishing intensity. Across scenarios, increasing fishing intensity in the absence of a constraining ecosystem cap results in stock and ecosystem overfishing and decreased biomass of groundfish predators.

Ecosystem caps on total fishery yield can be a tool to manage multispecies fisheries with an ecosystem approach (Link, 2018; Patrick & Link, 2015) and limit ecosystem overfishing (Link & Watson, 2019; Morrison et al., 2024). In Thailand, multispecies total allowable catch is set to 95% of the aggregate MSY of their marine resources, which, together with other measures, has had beneficial effects limiting overfishing (Kulanujaree et al., 2020). In a comparison of the fisheries management systems of the Bering Sea and the Northeast US, Link (2018) highlights that the one key aspect contributing to meeting management objectives in the former is the use of an aggregate portfolio approach to the OY cap. The GOA food web features top‐down dynamics (Adams et al., 2022; Barnes et al., 2020; Gaichas et al., 2015), and it is possible that deriving catch limits using single‐species approaches in this predation‐driven ecosystem led to overestimating the ecosystem cap. Our results suggest that, for ecosystem caps to be effective tools for ecosystem‐based fisheries management, they should be reviewed and adapted over time as environmental conditions, stock productivity, or species interactions change.

### Ecosystem caps in the context of total stock production

Our results support ecosystem caps below the sum of single‐species MSY because single‐species MSY neglects the production of a stock that is lost to predation and competition (Tyrrell et al., 2011). Such lost production can be large in a system with strong top‐down controls such as the GOA. Mueter and Megrey (2006) applied aggregate surplus production models and estimated a total groundfish production of ~2.5 million mt for the Bering Sea, compared to the 2 million mt OY cap for that region, and ~330,000 mt for the GOA, compared to the 800,000 mt cap. Similar models for other temperate ecosystems in the northern hemisphere (including Europe and North America) have shown that aggregate fishery production is ~75% of the sum of single‐species MSY estimates (Fogarty et al., 2012; Gaichas et al., 2012; Link et al., 2012; Lucey et al., 2012), with similar results from the Gulf of Thailand (Fulton et al., 2022). Because aggregate modeling approaches such as that of Mueter and Megrey (2006) implicitly represent losses due to predation, they may estimate more plausible removal limits within a top‐down controlled system such as the GOA than limits based on single‐species catch at *F*_MSY_ estimates. This is true *a fortiori* for ecosystem models that explicitly represent predator–prey interactions.

Estimates of maximum groundfish catch at equilibrium in Atlantis were generally higher than the estimates from Mueter and Megrey (2006), who estimated GOA‐wide multispecies surplus production to be 286 kt (146–445 kt 95% CI) or 332 kt (161–598 kt 95% CI) depending on the model used. One possible reason for this discrepancy is the release from predation of young spawners in our model, which explicitly captures trophic interactions. Age‐selective fishing releases younger age classes from top‐down control. Some GOA groundfish stocks (e.g., flatfishes other than halibut) are caught primarily as bycatch of other fisheries, and selectivity is skewed toward larger fish. As such, several functional groups in the model had at least one mature age class that was only partially targeted by fishing. While this is reasonable for GOA flatfishes, it can lead to stocks maintaining productivity even under high fishing mortality (Briton et al., 2019; Travers‐Trolet et al., 2020), which was observed to a certain extent in our analyses.

Maximum total groundfish catch in Atlantis was close to aggregate estimates of single‐species near‐term catch at *F*_MSY_ for the early 1990s, which is the reference period for groundfish biomass calibration in this model (Rovellini et al., 2024). Even so, aggregate yield never reached the OY cap, regardless of climate regime or arrowtooth exploitation level. We selected the 11 non‐halibut focal groups as the stocks accounting for most of the groundfish catch and because no estimates of *F*_MSY_ or its proxies exist for other groundfish stocks. If all other groundfish species were also exploited comparably, the OY cap could be met at least under simulated historical climate. However, given historical landings and current market values, it is unlikely that these non‐focal stocks will be exploited at higher levels in the future.

Groundfish bycatch (excluding Pacific halibut) must be accounted for when comparing aggregate yield to the OY cap. Our model captures all fishing mortality, including bycatch, with a single *F* per species. Modeling fleet‐specific fishing would allow us to explore how different fishing configurations may result in varying levels of bycatch and how this would impact the aggregate yield.

### Ecosystem caps depend on climate and trophic interactions

Multispecies yield is influenced by the effects of climate and predation pressure on targeted species. First, decreased groundfish yield in the projected climate change scenario was largely caused by the collapse of Pacific cod that resulted from the negative effects of temperature on spawning and recruitment (Laurel et al., 2023; Laurel & Rogers, 2020). The observed decline in Pacific cod yield under warm conditions was consistent with historical declines in Pacific cod catches during past heat waves (Barbeaux et al., 2020) and with projected declines of this species under future climate change (Holsman et al., 2020; Laurel et al., 2023; Punt et al., 2024). The slightly positive effects of projected climate change on the catches of most other focal groups were driven by consumption‐mediated increases in growth under ocean warming (Audzijonyte et al., 2020 Laurel et al., 2016; Oke et al., 2022), although this effect was reduced by lower food availability under reduced plankton productivity (Reum et al., 2024).

Second, arrowtooth is a key predator in the GOA and is responsible for high proportions of pollock natural mortality (Adams et al., 2022; Barnes et al., 2020; Gaichas et al., 2015). Predation from arrowtooth has also been linked to increased variability in GOA pollock mortality through time (Dorn & Barnes, 2022). Lighter fishing on arrowtooth resulted in higher arrowtooth biomass, which translated into higher predation pressure on other stocks and therefore lower catches. Such increased predation mortality when arrowtooth is underexploited has also been found in Bering Sea climate‐linked fishery simulations, where arrowtooth are important predators of pollock and Pacific cod (Reum et al., 2020). However, higher trophic‐level groundfish such as halibut and sablefish were less affected by arrowtooth top‐down control, highlighting that not all stocks provide higher yield under arrowtooth exploitation. Arrowtooth biomass in the GOA has declined over the past 10 years (Shotwell et al., 2023), which suggests that the top‐down control of this stock on other GOA groundfish may have been reduced. The effects of arrowtooth underexploitation on the system may be smaller than those observed in this study should arrowtooth biomass or productivity decline further in the future.

Arrowtooth is not the only important groundfish predator in the GOA ecosystem. Simultaneously increasing fishing mortality on focal groups other than arrowtooth led to increased groundfish biomass relative to simulations where *F* was manipulated on one focal group at a time. This suggests that other important groundfish predators (e.g., older Pacific cod, halibut, and sablefish) may produce “arrowtooth‐like” top‐down effects, especially when their consumption dynamics are synchronous in time or space (Barnes et al., 2020).

### Broader ecosystem impacts of multispecies fishing

Our results show that fishing intensity, climate regime, and arrowtooth exploitation level have implications for species other than the groundfish focal groups, including species of high conservation interest, via trophic interactions. Endangered Steller sea lions suffered from reduced total prey availability due to intensive groundfish fishing. Conversely, forage fish and some of their predators (dolphins and porpoises) benefited from the decline of predatory groundfish under increasing fishing mortality. However, arrowtooth underexploitation reduced such benefits for capelin, sand lance, and herring, which are important components of the arrowtooth diet (Barnes et al., 2021; Doyle et al., 2018; Gunther et al., 2024). Furthermore, benefits to forage fish, as well as dolphins and porpoises, from reduced groundfish predation on forage fish were eliminated by larger negative impacts of projected climate change. Such impacts, most likely due to decreased prey quality and quantity, have been observed in the GOA during recent marine heatwaves (Arimitsu et al., 2021; Baker et al., 2019; Suryan et al., 2021; von Biela et al., 2019).

Indirect effects of fishing on top predators varied depending on the specified reliance of each predator on groundfish prey in the model (e.g., high for pinnipeds and low for dolphins and porpoises). Predator biomass was lower under projected climate change conditions. This response was due to changes in prey availability because direct bioenergetic effects of temperature on endotherms are not represented in the model. Arrowtooth underexploitation resulted in Steller sea lions increasing their proportional uptake of this species, which partially offset the negative effects of decreasing prey availability. While Steller sea lions consume arrowtooth in some regions of the GOA (Sinclair & Zeppelin, 2002), this potential buffering effect of arrowtooth on sea lion biomass may be overly optimistic. Benthic‐foraging pinnipeds tend to exhibit slower population growth rates, potentially because of physiological constraints on diving capabilities, particularly for juveniles (Costa et al., 2004). An increase in benthic prey consumption by Steller sea lions in the GOA following the 2014–2016 marine heatwave coincided with reduced pup production and non‐pup counts (Maniscalco, 2023), which may have been due to reductions in prey energy density (von Biela et al., 2019) and/or overall abundance (Arimitsu et al., 2021) of prey, including forage fish. While pinnipeds feed on forage fish in the GOA (Sinclair & Zeppelin, 2002), Atlantis GOA did not capture a substantial shift to these prey species (except Pacific herring) as an alternative to groundfish. This is likely because the parameterization of their diet composition or a mismatch in the spatial overlap with their prey in the model limited their ability to do so.

Similar ecosystem‐level impacts of fishing have been shown in Walters et al. (2005), where fishing all species at single‐species MSY estimates led to detrimental effects on other trophic levels. We argue that effective ecosystem caps should account for interactions of target species with prey and predators that are not part of the fishery. Precautionary management measures have been introduced in Alaska to prevent excessive removals of groundfish prey for endangered Steller sea lions (Witherell, 2000), but the OY cap does not explicitly consider predator consumption needs. Our results emphasize that ecosystem models that explicitly capture predation are useful to evaluate the cascading effects of fishing on the whole food web by linking target species with other trophic levels.

### Caveats and potential future studies

While our simulations indicate that GOA groundfish productivity likely cannot support total catches exceeding the 800,000 OY cap, providing alternative estimates of the cap was beyond the scope of this study. The original value of the OY cap was derived numerically based on single‐species MSY considerations representative of the 1980s (Witherell, 2000), but the legal definition of OY in the US emphasizes that socioeconomic considerations are to be included in its determination (Restrepo et al., 1998). Future applications of Atlantis GOA could test the effects of imposing a range of values of the cap in a simulation framework. Such an approach would allow for evaluating the tradeoffs emerging from the application of an ecosystem cap and for testing the response of the system to realistic future fishing scenarios under climate change. Similarly, future applications could account for other important features of GOA fisheries, including gear types and spatial and seasonal closures.

The deterministic nature of Atlantis means that model results are a realization of the selected input parameters and that stochastic processes are not represented. Stochastic events, such as high or low recruitment, influence groundfish dynamics in Alaska (Barbeaux et al., 2020) and may affect how these species will respond to future climate change. While we evaluated end‐of‐run variability of selected quantities, future work should focus on conducting a more thorough analysis of uncertainty by running the model with alternative parametrizations and sets of forcings or initial conditions (e.g., McGregor et al., 2020). Additionally, comparing our results to those of Mueter and Megrey (2006) highlighted the value of using multiple modeling approaches to evaluate ecosystem caps. Model uncertainty could be addressed in future studies by evaluating the GOA OY cap using other models, such as Ecopath with Ecosim (EwE, Christensen & Walters, 2004) or *mizer* (Scott et al., 2014). EwE applications for the GOA exist (Gaichas et al., 2015) and may be built upon to address ecosystem cap questions such as those investigated here.

We applied forcings for ssp585, which is the highest CO_2_ emission scenario. There is increasing evidence that this set of conditions is unlikely, and less extreme warming is expected for the 21st century, albeit with large uncertainties (Burgess et al., 2023; Cooper et al., 2024). Testing the effects of more moderate climate emissions would likely result in less extreme effects. However, since total groundfish catch failed to meet the OY cap even under historical climate, especially under arrowtooth underexploitation, our main conclusions would be unchanged. Furthermore, our climate forcings did not represent pulse perturbations such as heatwaves, which are expected to become more frequent in the future in this region (Hicke et al., 2022) and whose effects on ecosystem productivity are difficult to predict.

We simplified some important aspects of fisheries management in the GOA for tractability and the purpose of focusing on OY. We modeled fishing with a time‐invariant *F*, irrespective of stock size. This allowed us to explore the response of the system to fishing pressures well above current limits, up to 4 *F*_MSY_. This approach forces the system into a state that it will not experience under current regulations. Groundfishes in the GOA are managed with single‐species harvest control rules that rescale *F* annually depending on stock status, ultimately closing the fishery in case of excessively depleted stocks (NPFMC, 2019).

## CONCLUSIONS AND MANAGEMENT IMPLICATIONS

Ecosystem caps in some regions of the world have shown promise as effective ways to maintain or increase food security and to address management mandates for optimizing catches. We showed that effective ecosystem caps must account for important aspects of multispecies systems, especially historical and future underutilization of fish stocks, predator–prey relationships, and linkages between ecosystem dynamics and future climate conditions. We showed how an end‐to‐end climate‐integrated ecosystem model can be used to generate ecological inferences for decision makers to set more effective ecosystem caps in a system with top‐down controls. We demonstrated how an ecosystem cap that has been used as a management tool in the GOA for over three decades but has never limited groundfish catches is unlikely to ever do so, especially under future climate change and arrowtooth underexploitation. Although the GOA ecosystem cap was a case study, this exercise shows that trophic interactions, climate conditions, and stock productivity should all be considered when identifying ecosystem caps. This may mean reviewing ecosystem caps when the structure and productivity of the ecosystem change, for example, in the case of events such as heat waves or regime shifts, species collapses, or substantial alteration of fishing patterns.

## CONFLICT OF INTEREST STATEMENT

The authors declare no conflicts of interest.

## Supporting information


Appendix S1.

## Data Availability

Data and code (Rovellini, 2025) are available on Zenodo at https://doi.org/10.5281/zenodo.15047964.
