# Supplementary material for: Evaluating ecosystem caps on fishery yield in the context of climate stress and predation
Source: Ecol Appl. 2025 May 12;35(3):e70036. doi: 10.1002/eap.70036 (PMC12069166; doi:10.1002/eap.70036)
Supplement: Supplementary file 1 — Appendix S1. [file EAP-35-e70036-s001.pdf]

Ecological Applications

Evaluating ecosystem caps on fishery yield in the context of climate stress and predation

Authors

Alberto Rovellini, André E. Punt, Martin W. Dorn, Isaac C. Kaplan, Meaghan D. Bryan, Grant Adams, Kerim Aydin, Matthew R. Baker, Cheryl L. Barnes, Bridget E. Ferriss, Elizabeth A. Fulton, Melissa A. Haltuch, Albert J. Hermann, Kirstin K. Holsman, Carey McGilliard, Elizabeth A. McHuron, Hem Nalini Morzaria-Luna, Szymon Surma

Appendix S1

Table S1. Parameter values for the unimodal bioenergetic response to temperature, corresponding to the temperature of optimum consumption (T_{C0}), the temperature at which consumption ceases (T_{CM}), and the ascending slope of the curve (Q_{10}). Formulation details are available in Rovellini et al. (2024).

**Species**	T_{C0} **(°C)**	T_{CM} **(°C)**	Q_{10}
Arrowtooth flounder	20.51	26	2.5
Big skate	12.11	17.3	2.36
Capelin	10	15	2.6
Deep demersal fish	8.09	11.56	2.36
Deep-water flatfish	12.11	17.3	2.36
Eulachon	10	15	2.6
Flathead sole	12.11	17.3	2.36
Forage fish - slope	10	15	2.6
Large sculpins	7.19	10.27	2.36
Longnose skate	12.83	18.32	2.36
Other skate	10.27	14.67	2.36
Pacific Ocean Perch	12.11	17.3	2.36
Pacific cod	13.7	21	2.41
Pacific hake	18.96	27.09	2.36
Pacific halibut	12.97	18	3.08
Pacific herring	10	15	2.6
Rex sole	12.11	17.3	2.36
Rockfish - demersal shelf	12.11	17.3	2.36
Rockfish - pelagic shelf assemblage	13.34	19.06	2.36
Rockfish - slope assemblage	8.88	12.68	2.36
Sablefish	12.11	17.3	2.36
Salmon chinook	13.34	19.06	2.36
Salmon chum	13.67	19.53	2.36
Salmon coho	18.96	27.09	2.36
Salmon pink	13.34	19.06	2.36
Salmon sockeye	8.96	12.8	2.36
Sand lance	10	15	2.6
Shallow demersal fish	14.2	20.29	2.36
Shallow-water flatfish	9.73	13.91	2.36
Thornyhead	19.05	27.22	2.36
Walleye pollock	10	15	2.6

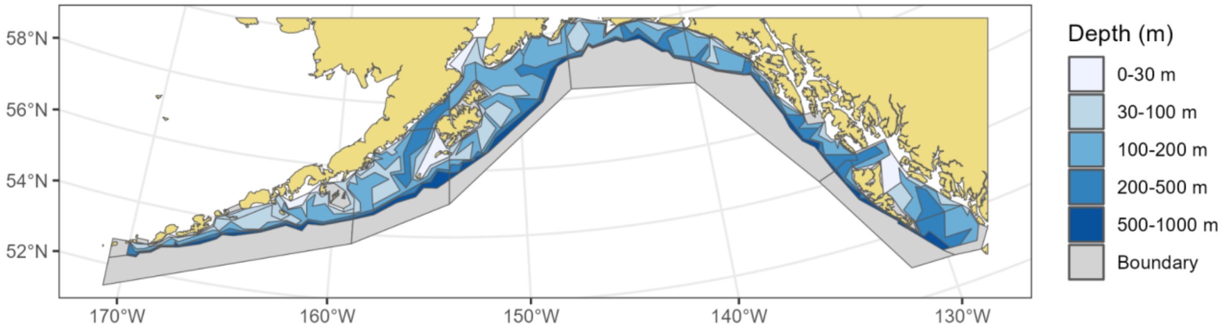

Figure S1. Model domain of Atlantis Gulf of Alaska. Grey boxes represent boundary boxes where biological and socioeconomic processes are not modeled dynamically.

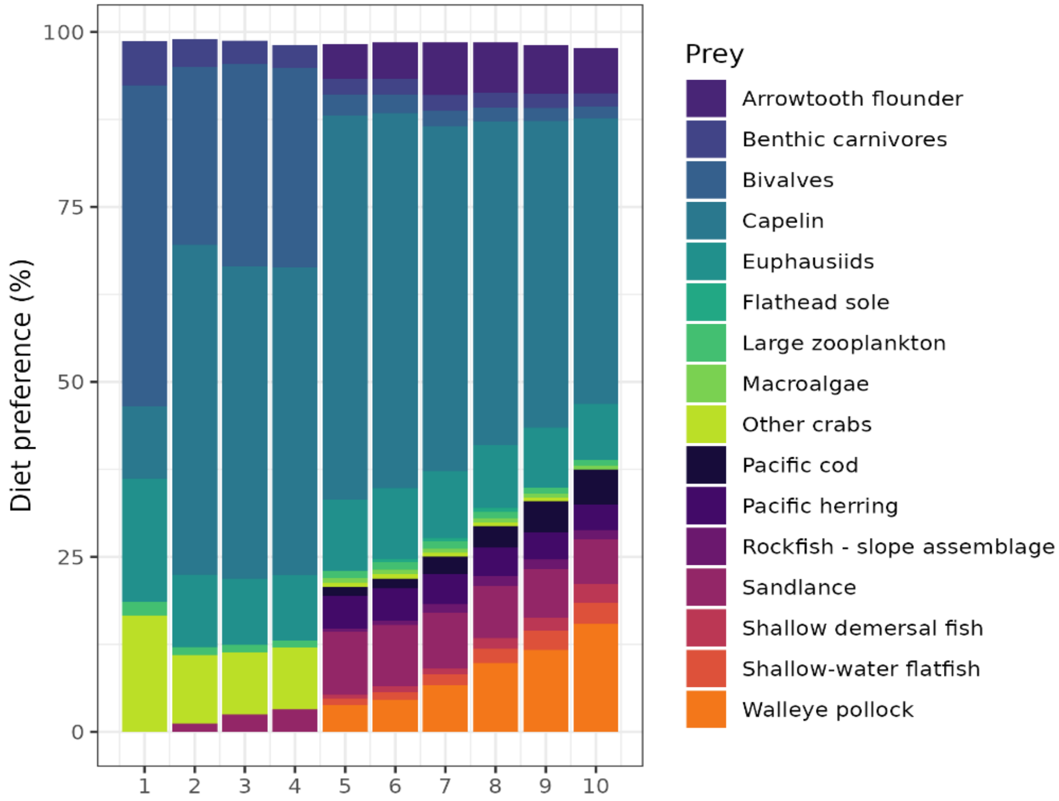

Figure S2. Diet composition at model equilibrium of arrowtooth flounder by age class (2 years per age class), for the calibrated Atlantis GOA base model. Means over the last 5 simulated years are shown, therefore no seasonality is captured in this plot. Bars do not add up to 100% because only prey accounting for >0.5% of the total consumption (by biomass) are plotted for ease of visualization.

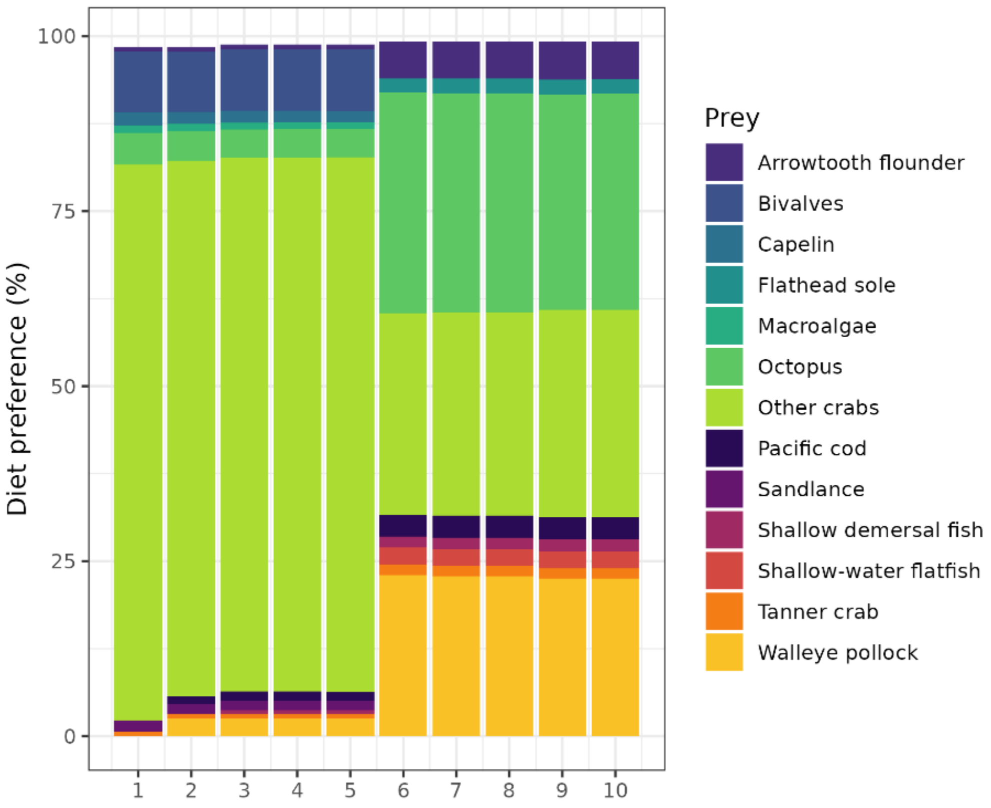

Figure S3. Diet composition at model equilibrium of Pacific halibut by age class (3 years per age class), for the calibrated Atlantis GOA base model. Means over the last 5 simulated years are shown, therefore no seasonality is captured in this plot. Bars do not add up to 100% because only prey accounting for >0.5% of the total consumption (by biomass) are plotted for ease of visualization.

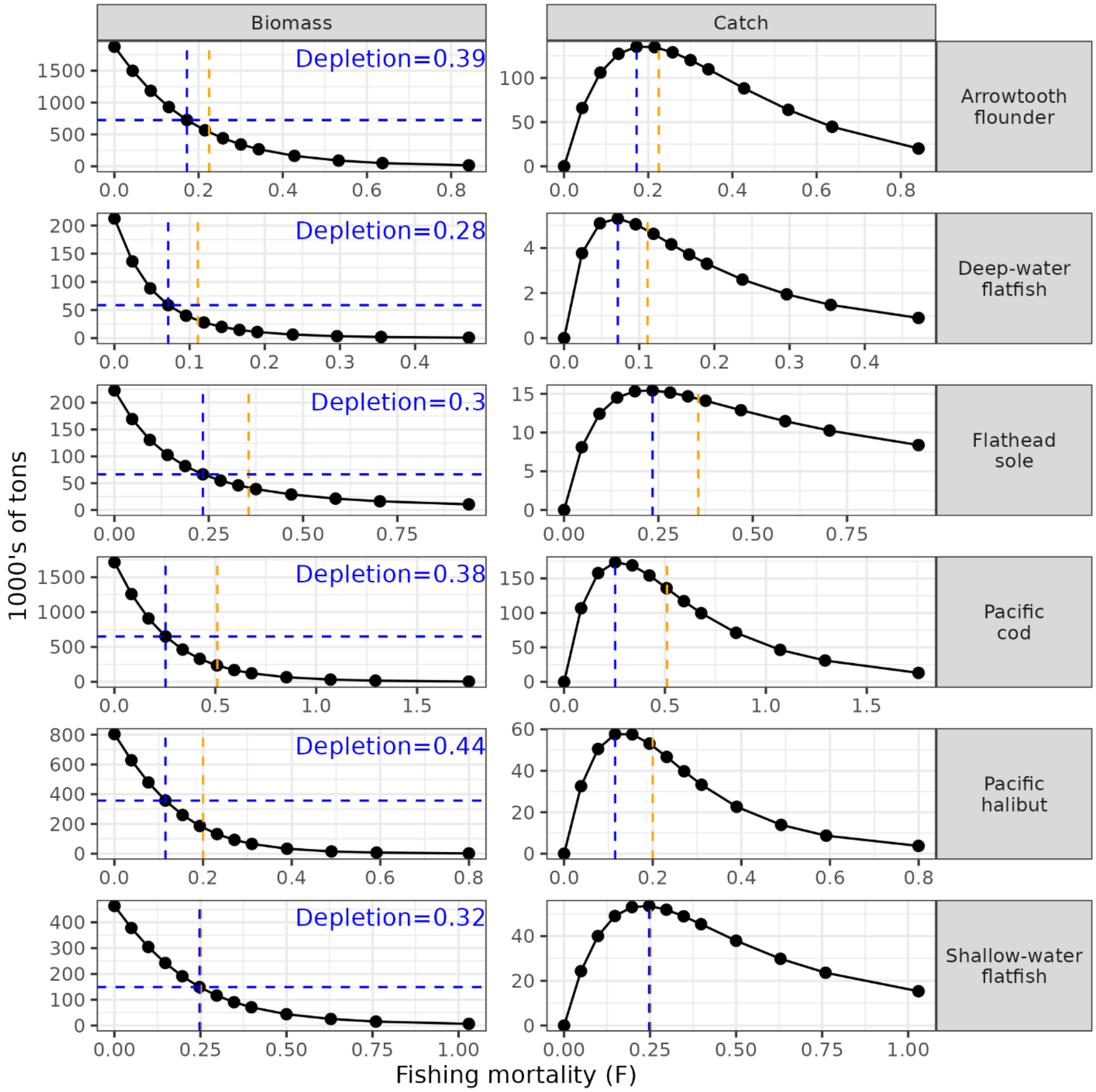

Figure S4. Equilibrium spawning biomass (left) and catch (right) in 1000s of tons of the groundfish focal groups as a function of fishing mortality, as determined in step one of the Atlantis fishing experiment (F manipulated on one focal group at a time). Vertical dashed lines: in orange, F_{OFL} from single-species assessments (M for Pacific halibut), and in blue F_{MSY} as determined by Atlantis. F_{OFL} is a proxy of F_{MSY} in Alaska management. Horizontal dashed lines indicate stock biomass at F_{MSY}, with the corresponding stock depletion.

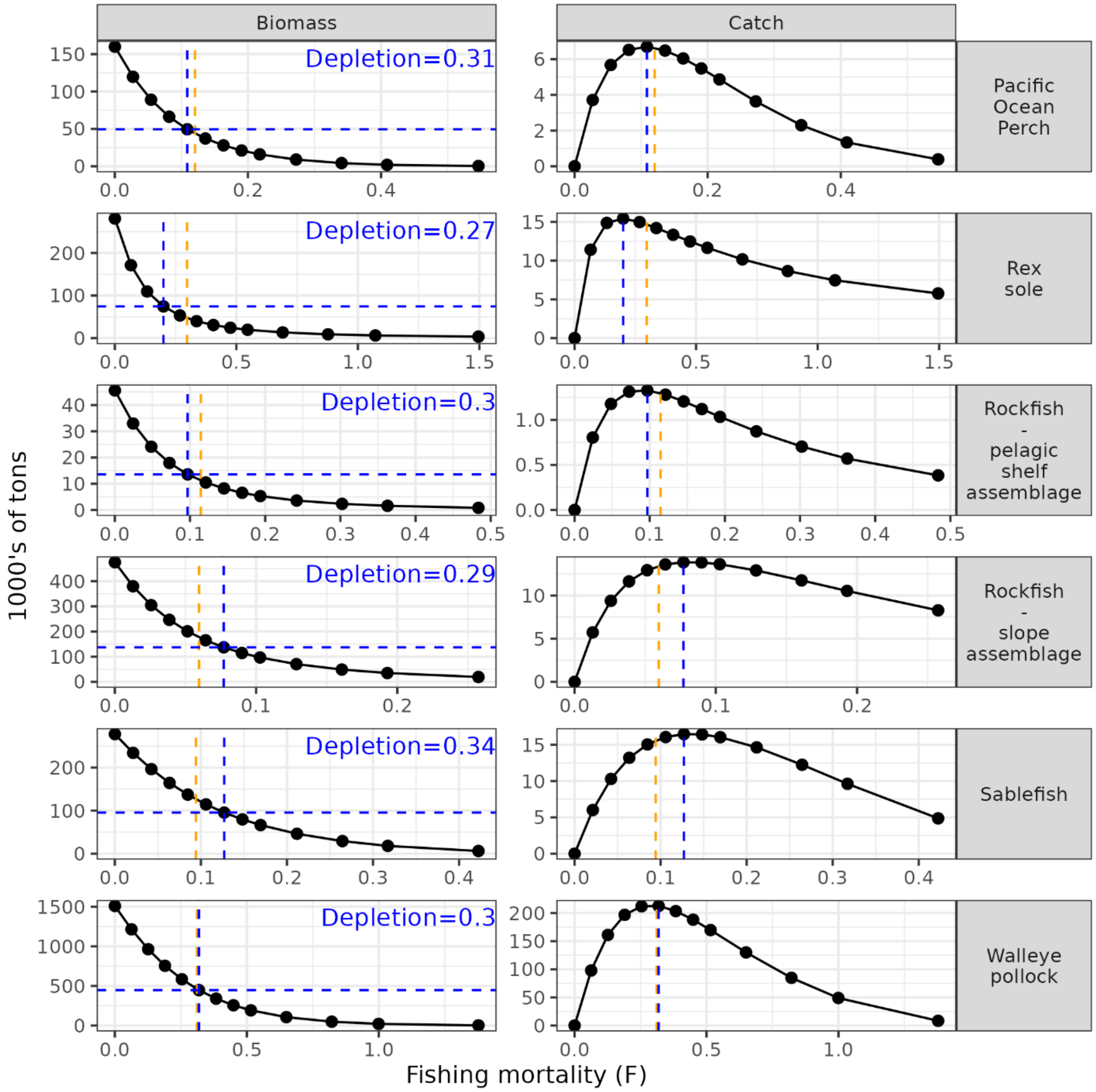

Fig. S4. Continued.

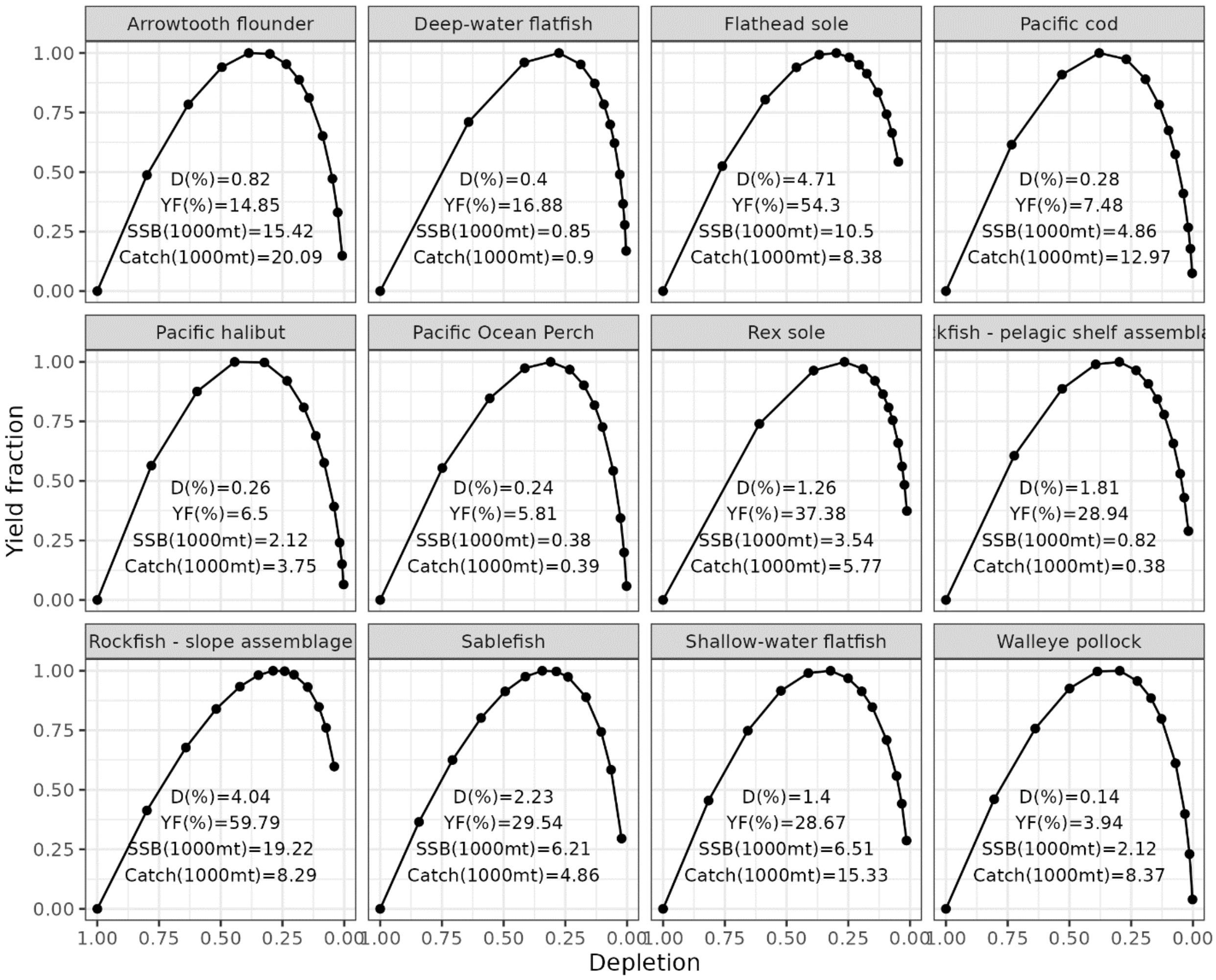

Figure S5. Production curves of the groundfish focal groups from step one of the Atlantis fishing experiment (F manipulated on one focal group at a time). Annotations refer to the following quantities under the highest fishing mortality tested ($F = 4F_{MSY}$): D = depletion, i.e. the fraction of unfished biomass; YF = yield fraction; SSB = spawning stock biomass; and catch.

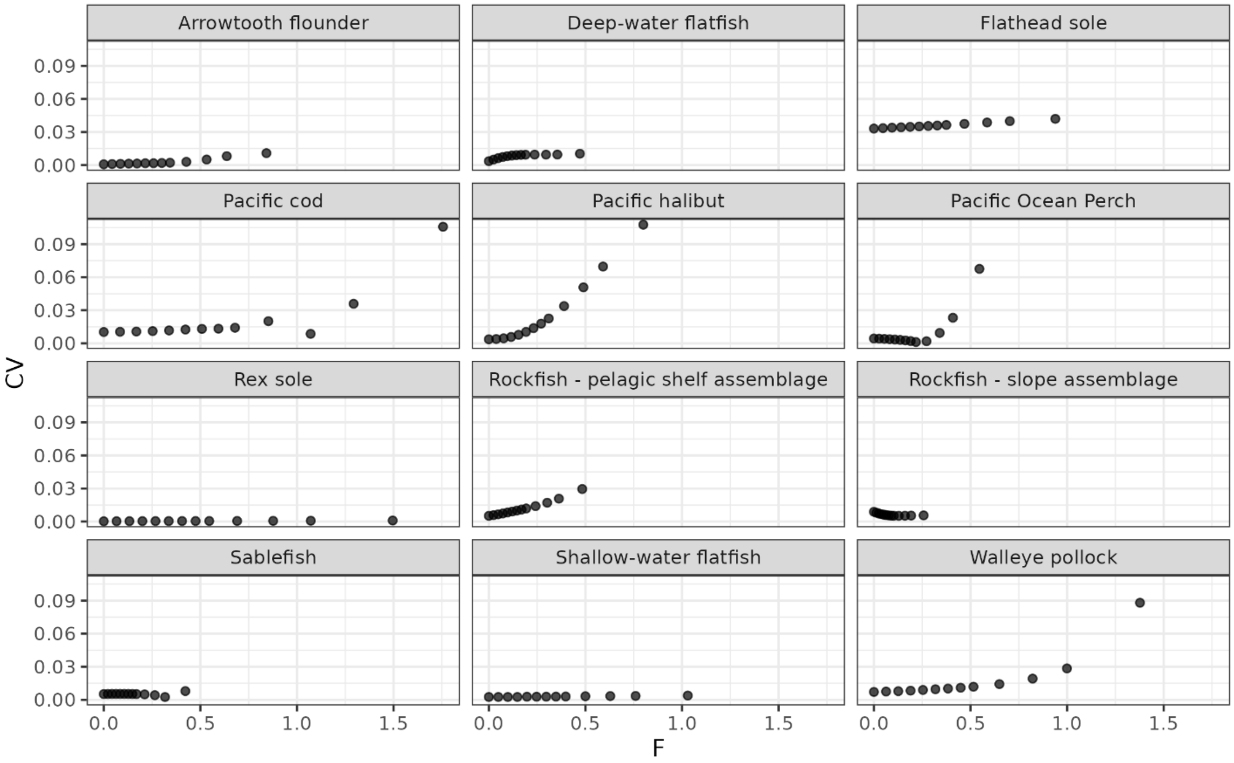

Figure S6. Coefficients of variation (CV) of the biomass of the 12 focal groups as a function of fishing mortality F, computed over the last 5 years of the model runs performed in step one. Results for catch CVs are almost identical and are therefore not shown.

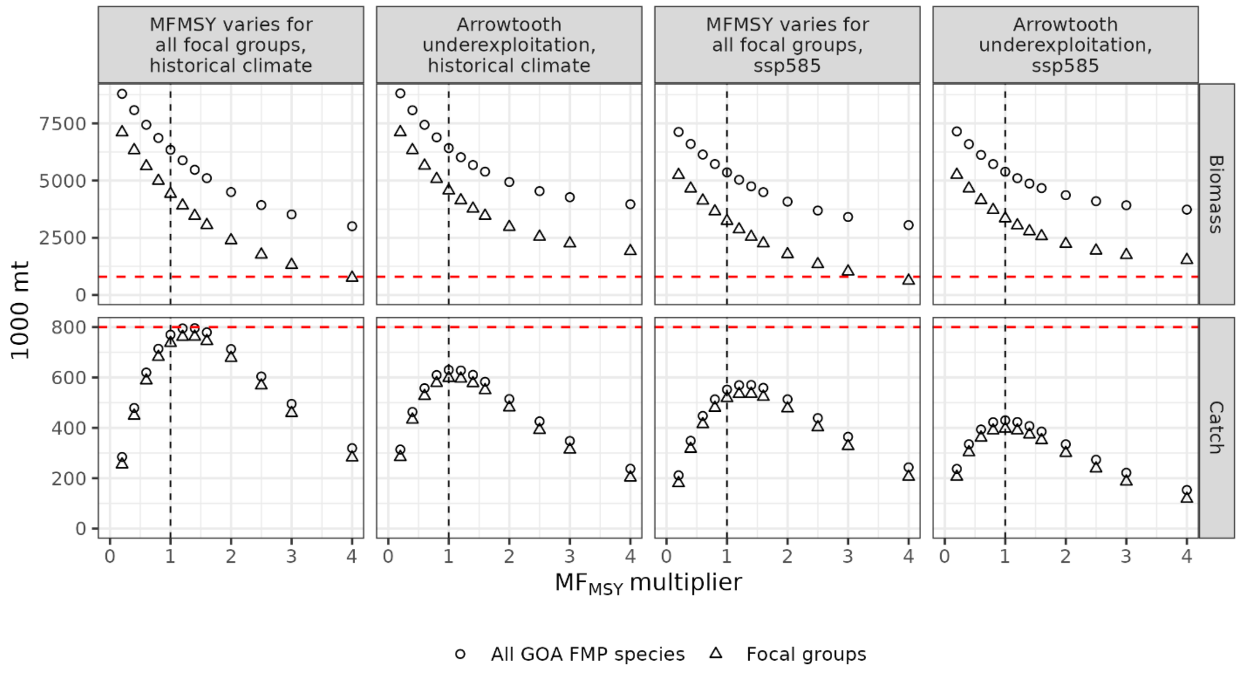

Figure S7. Equilibrium multispecies groundfish biomass (top) and yield (bottom) in 1000s of tons for increasing multipliers of the multispecies fishing mortality at maximum sustainable yield (MF_{MSY}). Circles indicate aggregate values of all groundfish species in the Gulf of Alaska groundfish Fishery Management Plan (FMP); triangles indicate aggregate values of the 11 non-halibut focal groups. Panels indicate the four scenarios combining climate regime and arrowtooth flounder exploitation level. Only catches from the Alaska portion of the model domain are shown, and Pacific halibut catches are not plotted, to allow comparison with the Gulf of Alaska optimum yield cap (horizontal red dashed line = 800,000 mt). The vertical dashed line indicates MF_{MSY}.

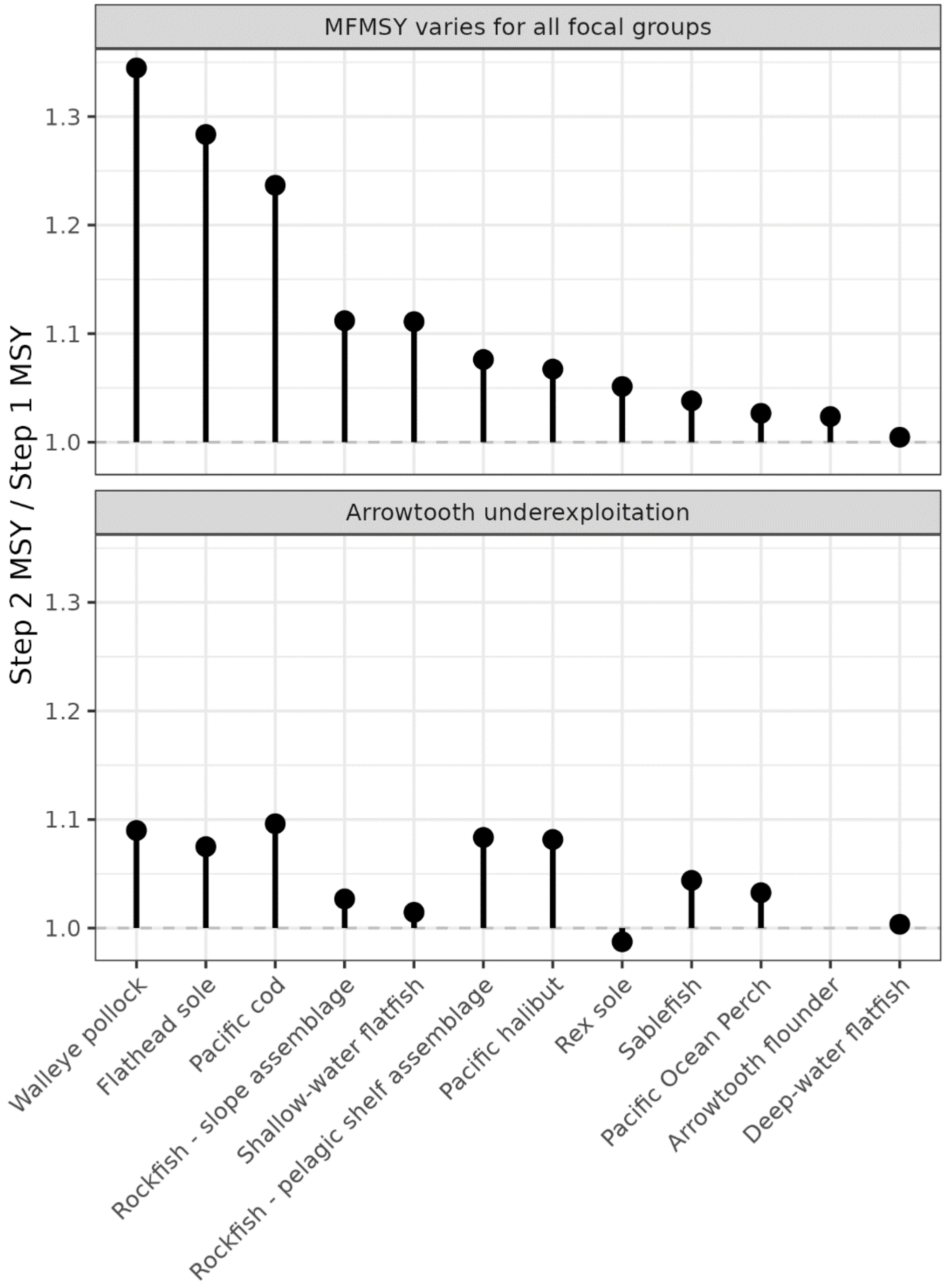

Figure S8. Top panel: Ratio between maximum sustainable yield (MSY) of each focal group calculated in Atlantis in steps two and one of the fishing experiment. In step one, fishing mortality (F) is manipulated for one focal group at a time, while in step two F is manipulated for all focal groups simultaneously. Bottom panel: same as top panel but for the scenario where arrowtooth is underexploited in step two (under historical climate conditions).

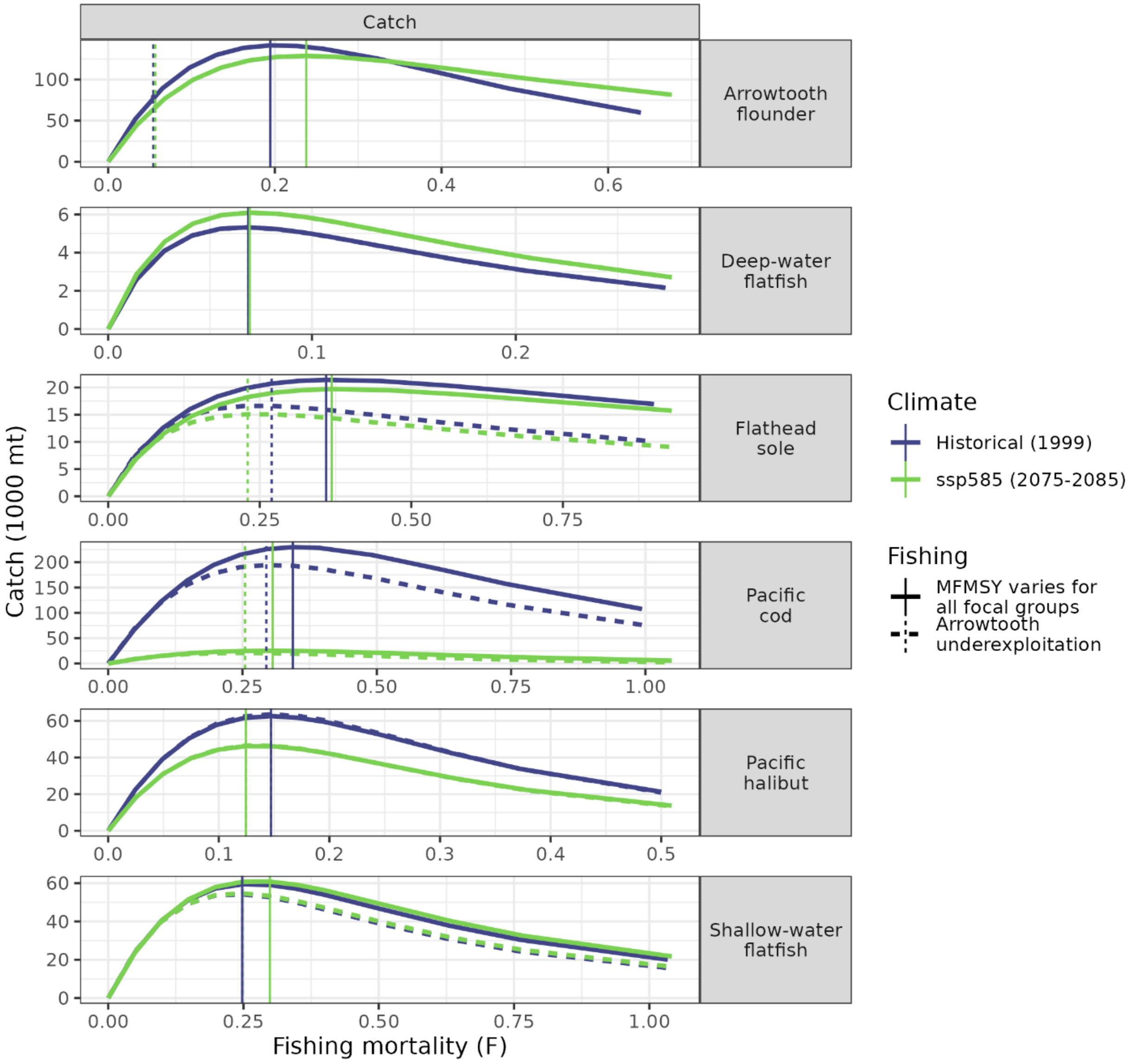

Figure S9. Equilibrium catch (1000s of tons) of the groundfish focal groups for increasing fishing mortality under different combinations of climate regimes and arrowtooth fishing strategy. Solid lines indicate that F on all stocks varies with the multipliers of MF_{MSY}; dashed lines indicate F on arrowtooth flounder fixed at ¼ F_{OFL}. Blue lines: historical conditions (1999); green lines: warm conditions (2075-2085 climatology, ssp585). Vertical lines indicate F corresponding to highest yield in each scenario.

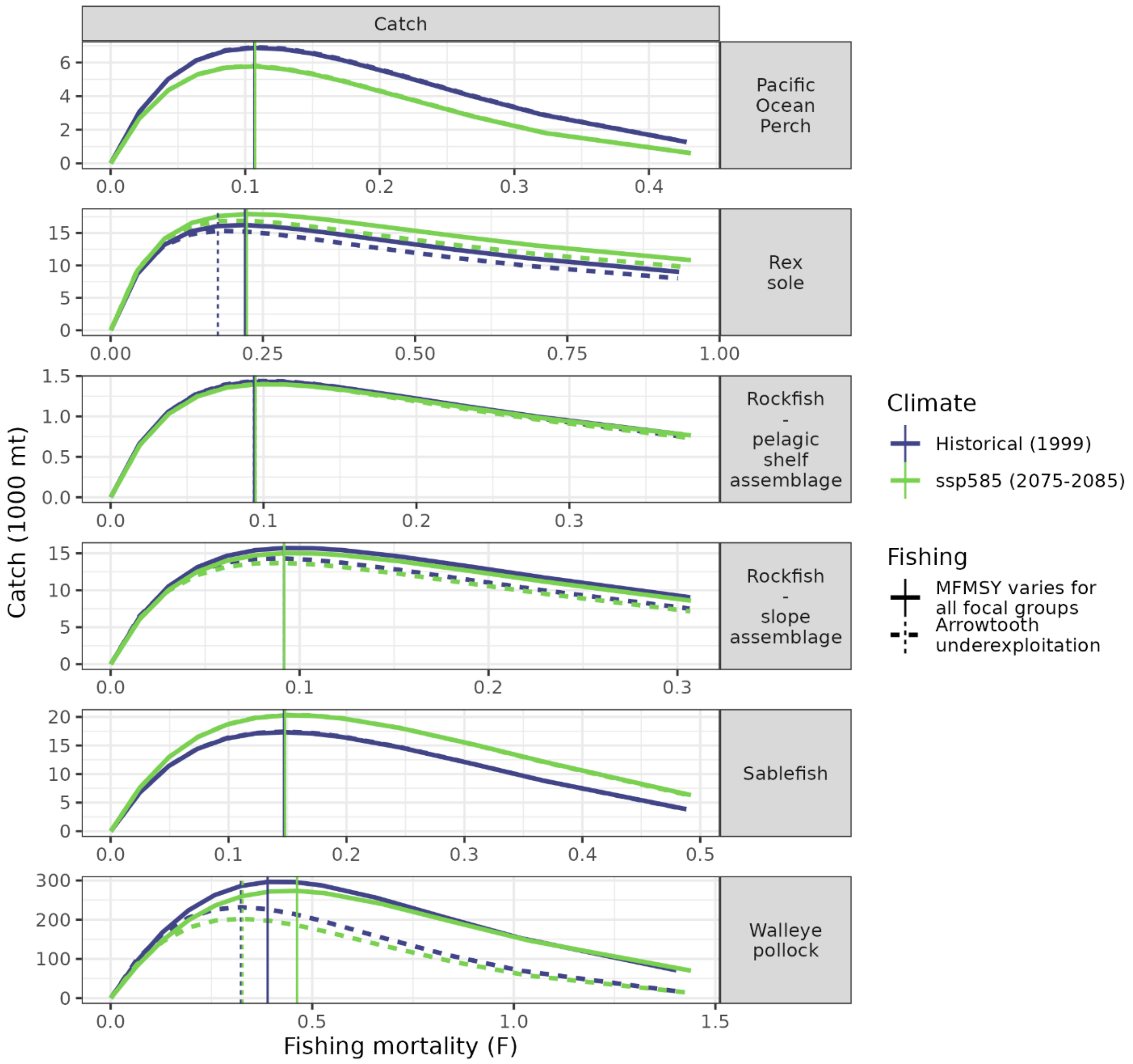

Figure S9. Continued.

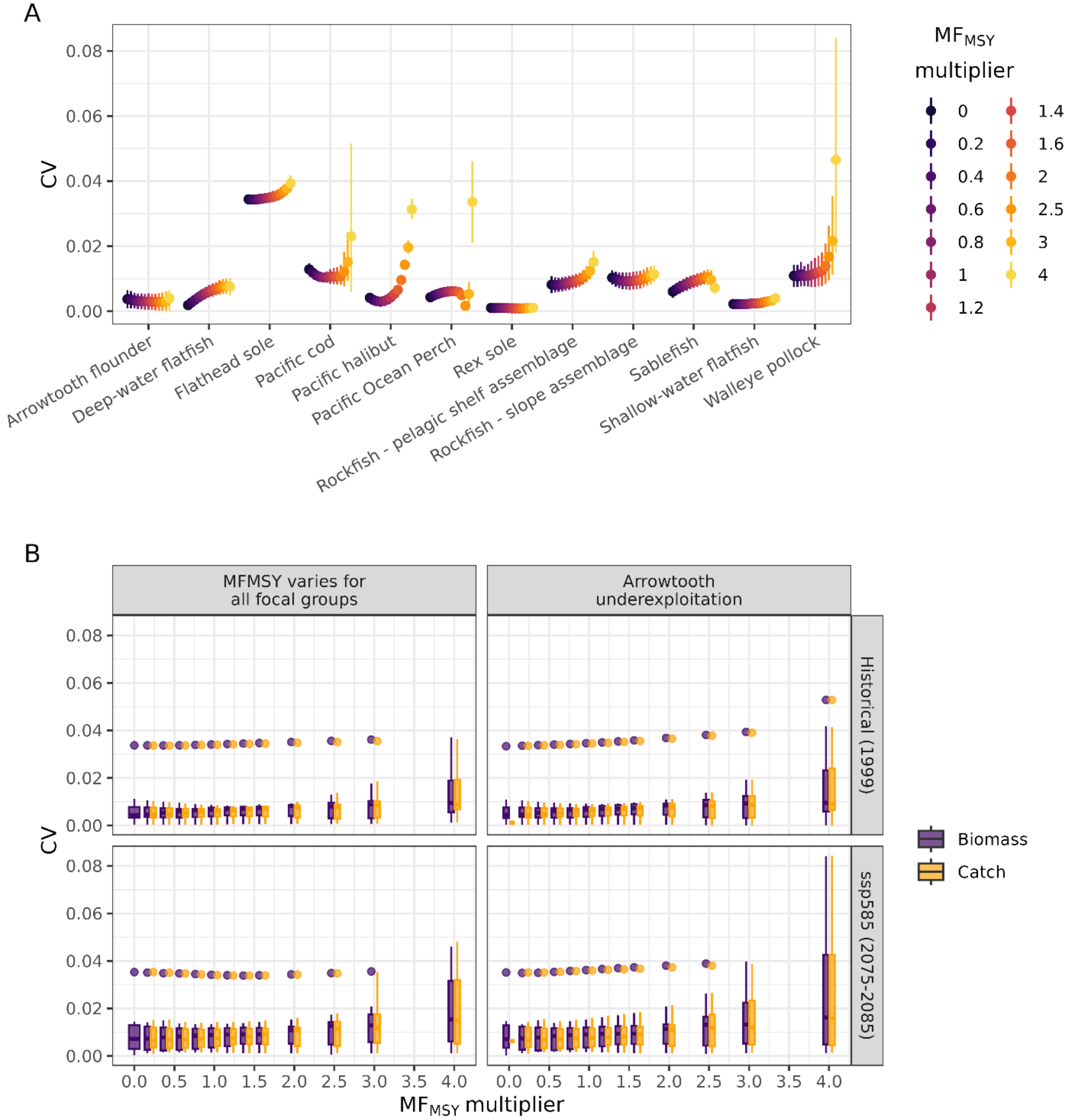

Figure S10. A: Coefficients of variation (CV) of the biomass of the 12 focal groups computed over the last 5 years of the model runs performed in step two. Points represent the mean values for the four scenarios, while bars span from the minimum to the maximum values. Colors indicate the MF_{MSY} multiplier. Results for the catch CVs are almost identical and are therefore not shown. B: Boxplots of the CVs of biomass (purple) and catch (orange) for the 12 focal groups over increasing MF_{MSY} multipliers and across modeled scenarios.

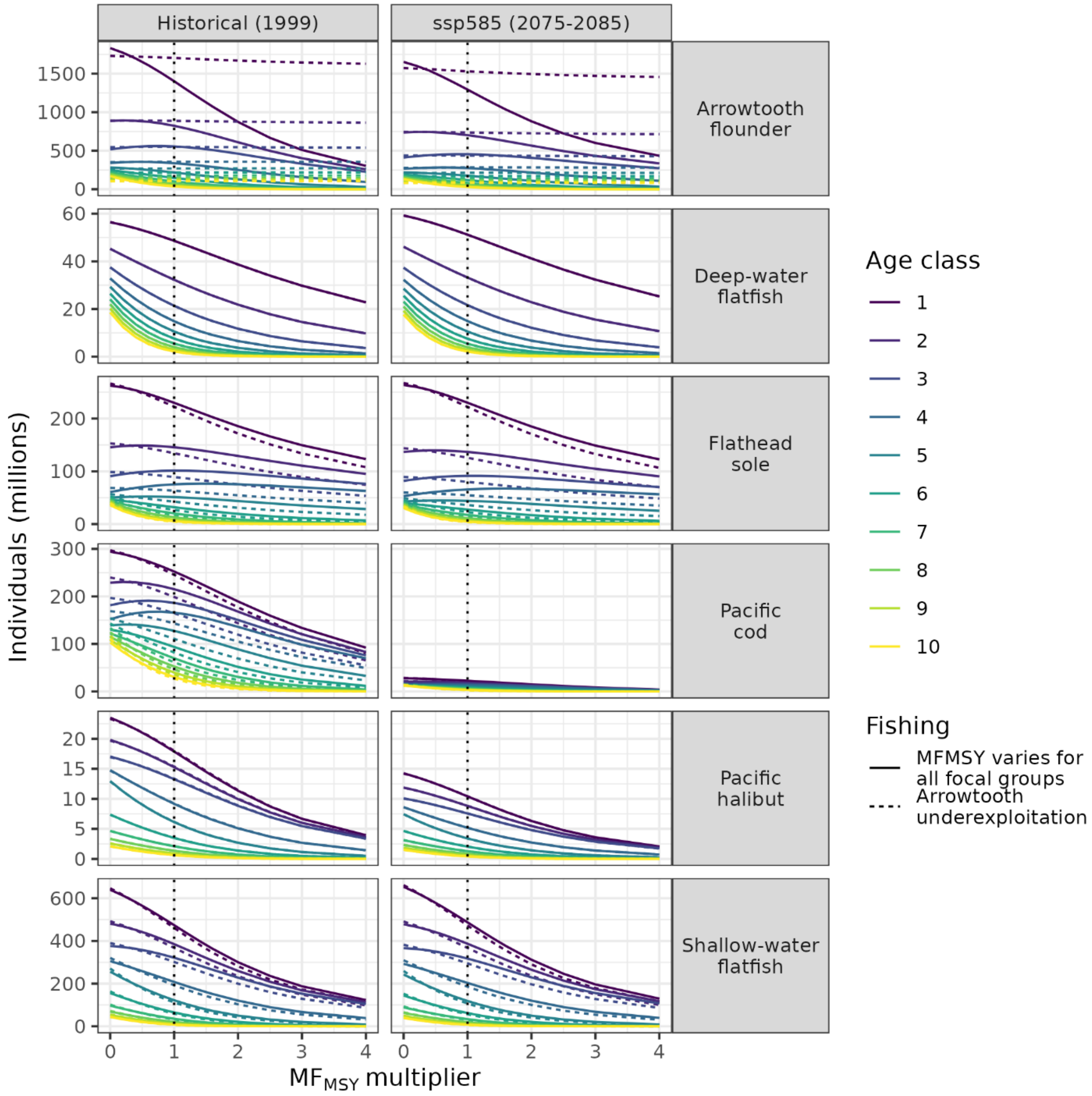

Figure S11. Numbers-at-age (millions) at equilibrium by Atlantis age-class (which may combine multiple annual cohorts) for the groundfish focal groups as a function of the MF_{MSY} multiplier. Columns indicate the climate regime, rows indicate the focal group. Solid lines indicate that F on all stocks varies with the multipliers of MF_{MSY}; dashed lines indicate F on arrowtooth fixed at ¼ F_{OFL}. Colors indicate age classes. The vertical dotted line indicates MF_{MSY}.

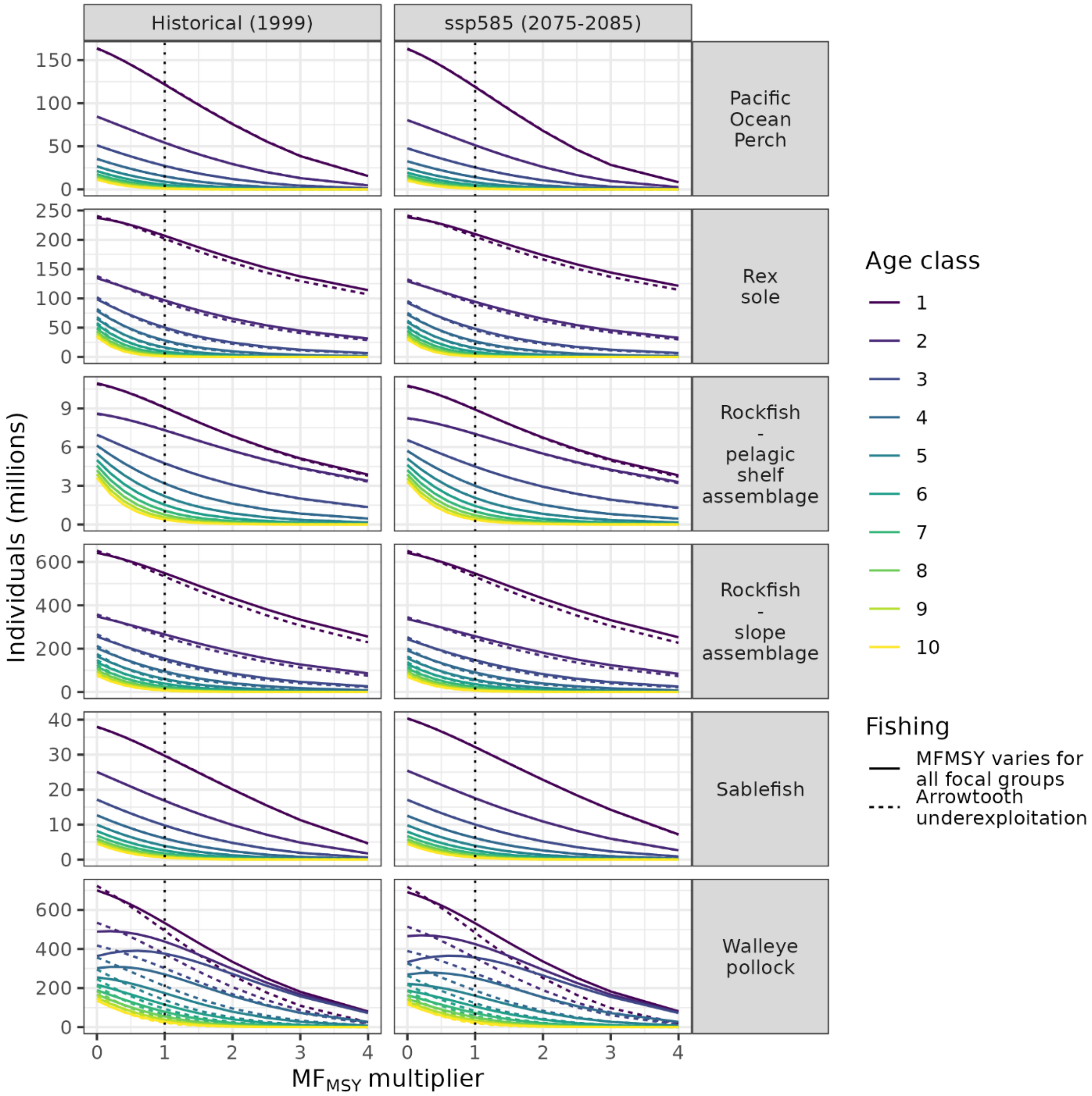

Figure S11. Continued.

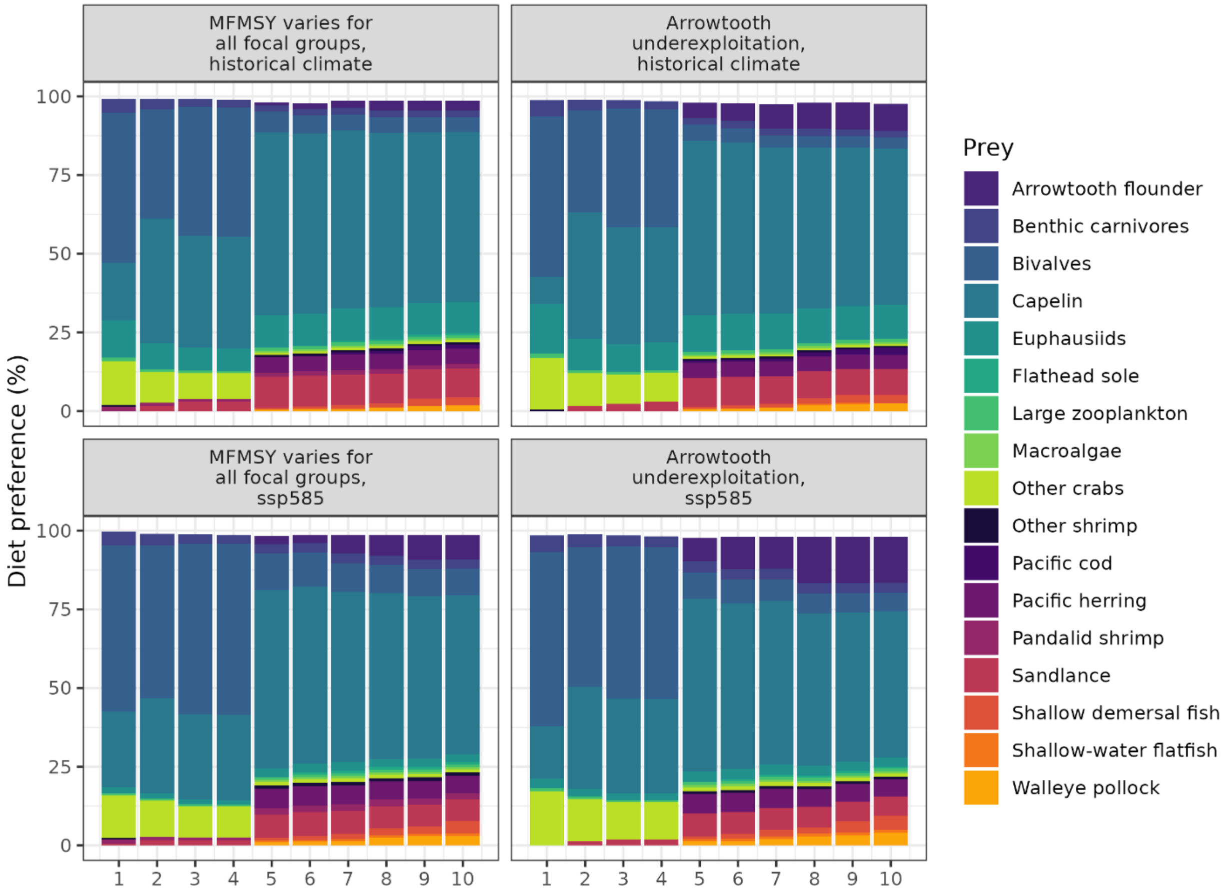

Figure S12. Diet compositions (in % by weight) at equilibrium by age class (2 years) of arrowtooth flounder in the Atlantis Gulf of Alaska model. Panels refer to different scenarios. All panels capture the highest fishing intensity for each scenario. Bars may not add up to 100% because only prey species accounting for >0.5% of the total consumption (by biomass) are plotted.

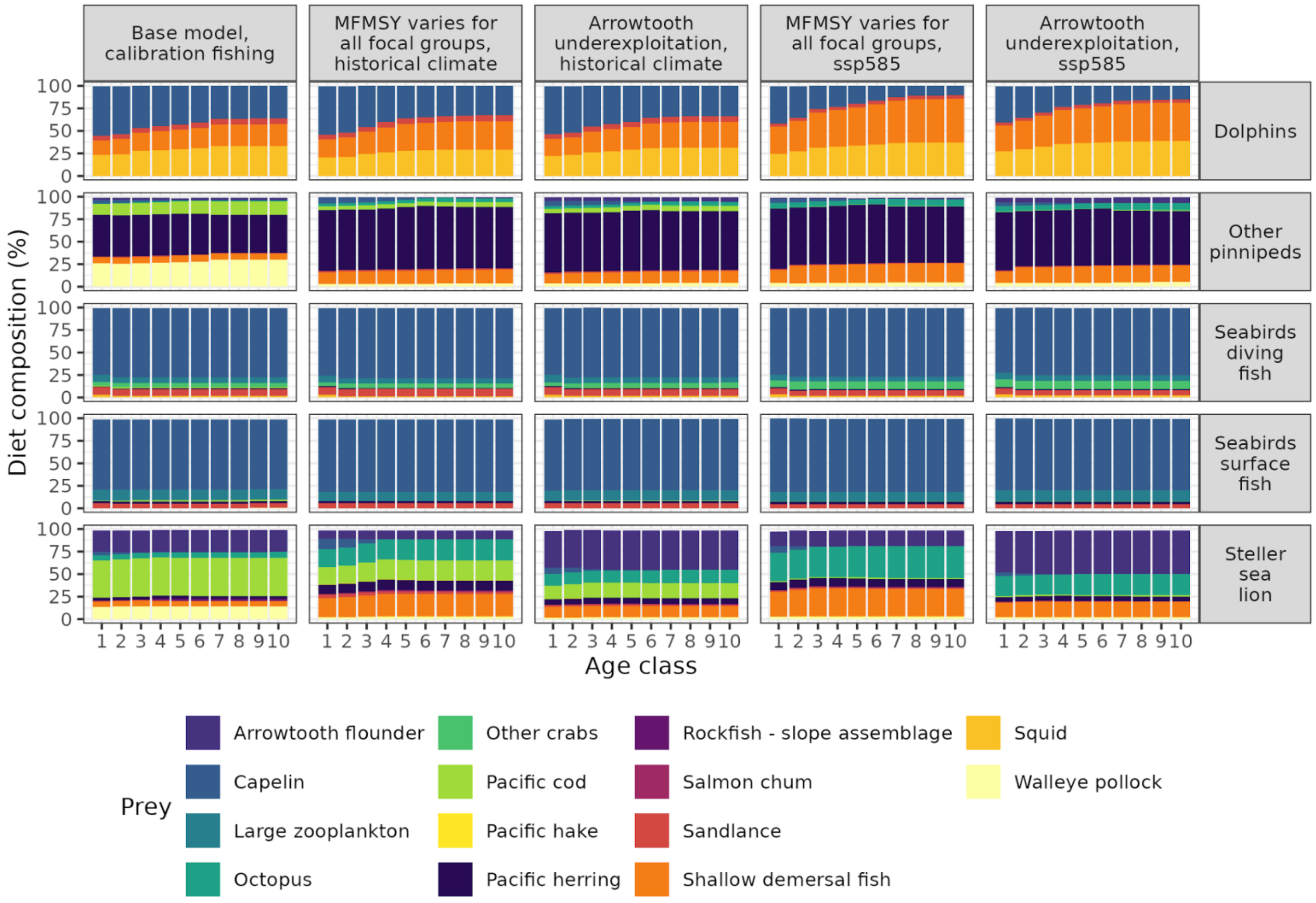

Figure S13. Diet compositions (in % by weight) at equilibrium by multi-year age class (2 years for all these groups) of selected groundfish predators in Atlantis GOA. Columns refer to different scenarios. All columns capture the highest fishing intensity for each scenario (except the leftmost column, which refers to the calibrated model with fishing at ¼ F_{OFL}). Rows are the predator functional groups, and colors of the bars are the prey species. Bars may not add up to 100% because only prey species accounting for >0.5% of the total consumption (by biomass) are plotted.

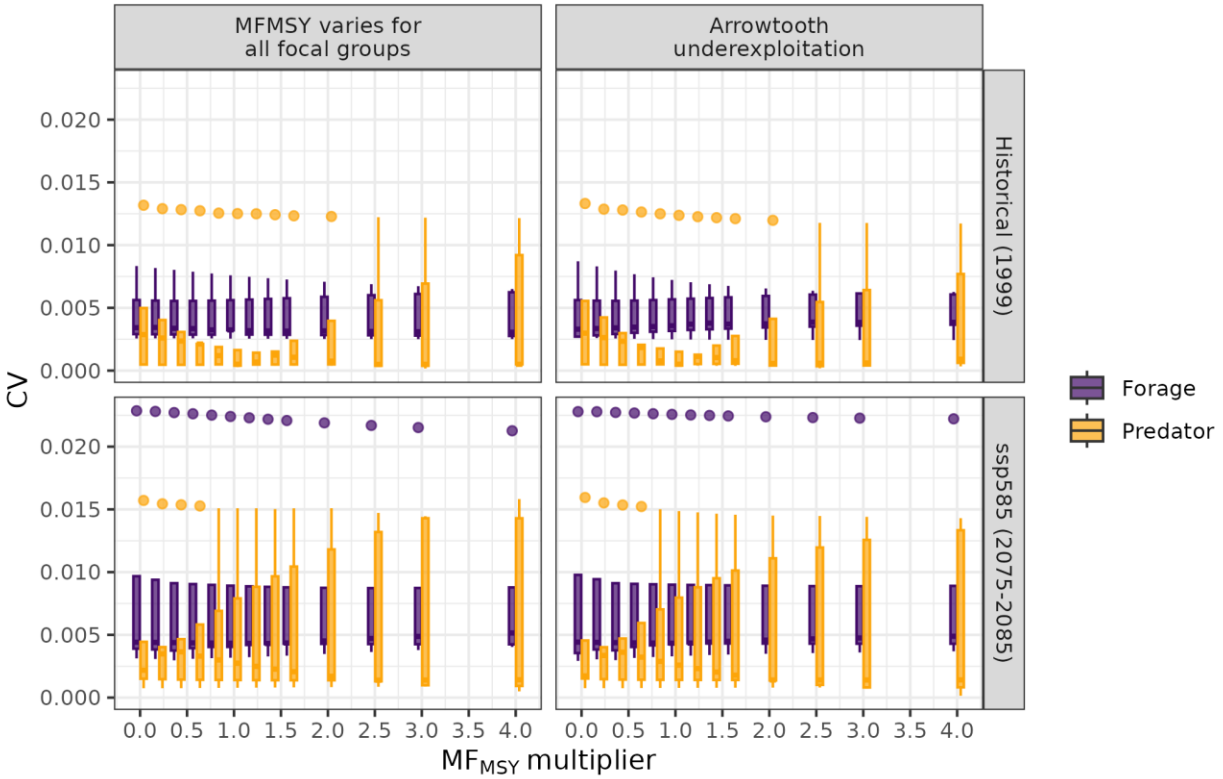

Figure S14. Boxplots of the coefficients of variation (CV) of the biomass of forage fish functional groups (capelin, Pacific herring, Pacific sand lance, eulachon, and slope forage fish) in purple, and of piscivorous predators (Steller sea lions, other pinnipeds, dolphins and porpoises, and seabirds) in orange for increasing MF_{MSY} multipliers on groundfish and across modeled scenarios.

Reference

Rovellini, A., Punt, A. E., Bryan, M. D., Kaplan, I. C., Dorn, M. W., Aydin, K., et al. (2024). Linking climate stressors to ecological processes in ecosystem models, with a case study from the Gulf of Alaska. ICES Journal of Marine Science, Volume 82, Issue 1. fsae002.
